# Transformation-Optics-Designed Plasmonic Singularities for Efficient Photocatalytic Hydrogen Evolution at Metal/Semiconductor Interfaces

**DOI:** 10.1021/acs.nanolett.3c01287

**Published:** 2023-05-26

**Authors:** Tingting Lin, Tianyi Yang, Yuhang Cai, Jingwei Li, Guangxiang Lu, Shuangqun Chen, Yi Li, Liang Guo, Stefan A. Maier, Changxu Liu, Jianfeng Huang

**Affiliations:** † State Key Laboratory of Coal Mine Disaster Dynamics and Control, Institute of Advanced Interdisciplinary Studies, School of Chemistry and Chemical Engineering, Chongqing University, Chongqing 400044, China; ‡ School of Physics and Astronomy, Monash University, Clayton, Victoria 3800, Australia; § Department of Mechanical and Energy Engineering, Southern University of Science and Technology, Shenzhen 518055, China; ∥ Centre for Metamaterial Research & Innovation, Department of Engineering, University of Exeter, Exeter EX4 4QF, United Kingdom; ⊥ School of Microelectronics, MOE Engineering Research Center of Integrated Circuits for Next Generation Communications, Southern University of Science and Technology, Shenzhen 518055, China; # Blackett Laboratory, Imperial College London, London SW7 2BZ, United Kingdom; □ Chair in Hybrid Nanosystems, Nanoinstitute Munich, Faculty of Physics, Ludwig Maximilians University of Munich, 80539 Munich, Germany; ○ Department of Mathematics, Physics and Electrical Engineering, Northumbria University, Newcastle Upon Tyne NE1 8ST, United Kingdom

**Keywords:** transformation optics, plasmonics, photocatalysis, hydrogen evolution, hybrid nanostructures

## Abstract

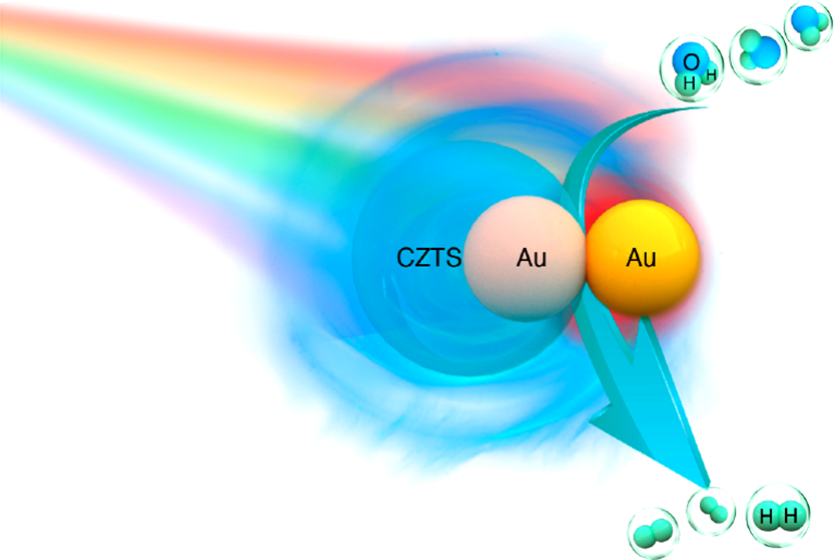

Inspired by transformation optics, we propose a new concept for plasmonic photocatalysis by creating a novel hybrid nanostructure with a plasmonic singularity. Our geometry enables broad and strong spectral light harvesting at the active site of a nearby semiconductor where the chemical reaction occurs. A proof-of-concept nanostructure comprising Cu_2_ZnSnS_4_ (CZTS) and Au–Au dimer (t-CZTS@Au–Au) is fabricated via a colloidal strategy combining templating and seeded growth. On the basis of numerical and experimental results of different related hybrid nanostructures, we show that both the sharpness of the singular feature and the relative position to the reactive site play a pivotal role in optimizing photocatalytic activity. Compared with bare CZTS, the hybrid nanostructure (t-CZTS@Au–Au) exhibits an enhancement of the photocatalytic hydrogen evolution rate by up to ∼9 times. The insights gained from this work might be beneficial for designing efficient composite plasmonic photocatalysts for diverse photocatalytic reactions.

Taking advantage of strong interaction with resonant photons through the excitation of localized surface plasmon resonance (LSPR), a collective and coherent oscillation of free electrons,^1^ plasmonic nanostructures have been utilized for a repertoire of photodriven chemical conversions,^2,3^ in terms of either directly triggering the photocatalysis^4−12^ or indirectly accelerating photocatalysis on neighboring semiconductors.^13−19^ Among the photocatalytic reactions, solar water splitting producing hydrogen typically on semiconductors has aroused particular interest owing to the contemporary urgent demand for developing renewable alternatives to fossil fuels.^20−23^ Despite impressive recent achievements in the fundamental understanding and practical performance improvement in plasmonic photocatalysis,^24,25^ most plasmonic photocatalysts employed regular plasmonic nanocrystals with only narrowband spectral light harvesting capabilities, limiting the exploitation of the full potential of SPR-mediated photocatalysis.^26−30^ The further promotion of current plasmonic catalytic systems is of paramount significance but remains challenging. It relies on a rational design of composite plasmonic-metal/semiconductor photocatalysts with favorable optical properties.

The exciting advances in transformation optics first proposed over a decade ago directed the design of singular nanostructures with unparalleled abilities to interact with photons covering a broad spectral range.^31−33^ Singular structures such as sharp tips and kissing points can concentrate photons to the geometric singularity, where gigantic electromagnetic field enhancement can be achieved.^34,35^ Consequently, singular features confer the nanostructures with a broadband response to electromagnetic fields,^36,37^ overlapping a major spectrum of solar radiation. Several transformation-optics-inspired applications in solar energy harvesting have been theoretically proposed^35,38,39^ and experimentally demonstrated.^37,40,41^ However, the ideas of transformation optics have not been directly exploited for photocatalysis experimentally.

Here, we aim to bridge the gap between these two research fields, realizing an efficient plasmonic photocatalyst for hydrogen evolution and offering an explicit strategy for singularity-assisted photocatalysis. We design and formulate transformation-optics-inspired heterogeneous asymmetric hybrid nanostructures (HNSs) for hydrogen evolution based on LSPR mediated photocatalysis. The proof-of-concept hybrid nanostructure (HNS) is composed of a Au nanoparticle-Au nanoparticle (Au NP-Au NP) dimer with an additional CZTS NP *tangentially* covering only one Au NP of the dimer (denoted as **t-CZTS@Au–Au** HNS) (Figure 1). The CZTS, a semiconductor possessing a direct and narrow band gap (∼1.5 eV) and comprising abundant and environmentally benign elements, operates as an active site for hydrogen evolution,^15,42,43^ while the Au–Au entity performs as a nanoharvester collecting the energy from visible and near-infrared photons to boost the photocatalytic efficiency via plasmonic enhancement. We achieve a record-high hydrogen evolution rate of ∼1192 μmol h^–1^ g^–1^ among reported plasmonic-metal/CZTS composites (Figure S1), by virtue of exquisite geometric control of the catalyst at the nanoscale with colloidal chemistry. Sharp contacts between Au NPs are produced for broadband energy harvesting and gigantic field enhancement through adiabatic focusing. Meanwhile, the precise placement of such hotspots to the metal/semiconductor/liquid interface can maximize the usage of energetic carriers for hydrogen generation, as proved by different control samples combined with *ab initio* full wave simulations. The strategy of applying the judicious optical design from transformation optics into photocatalysis sheds light on the production of clean and renewable energy or valuable products from solar radiation.^26,27,44,45^

**Figure 1:**
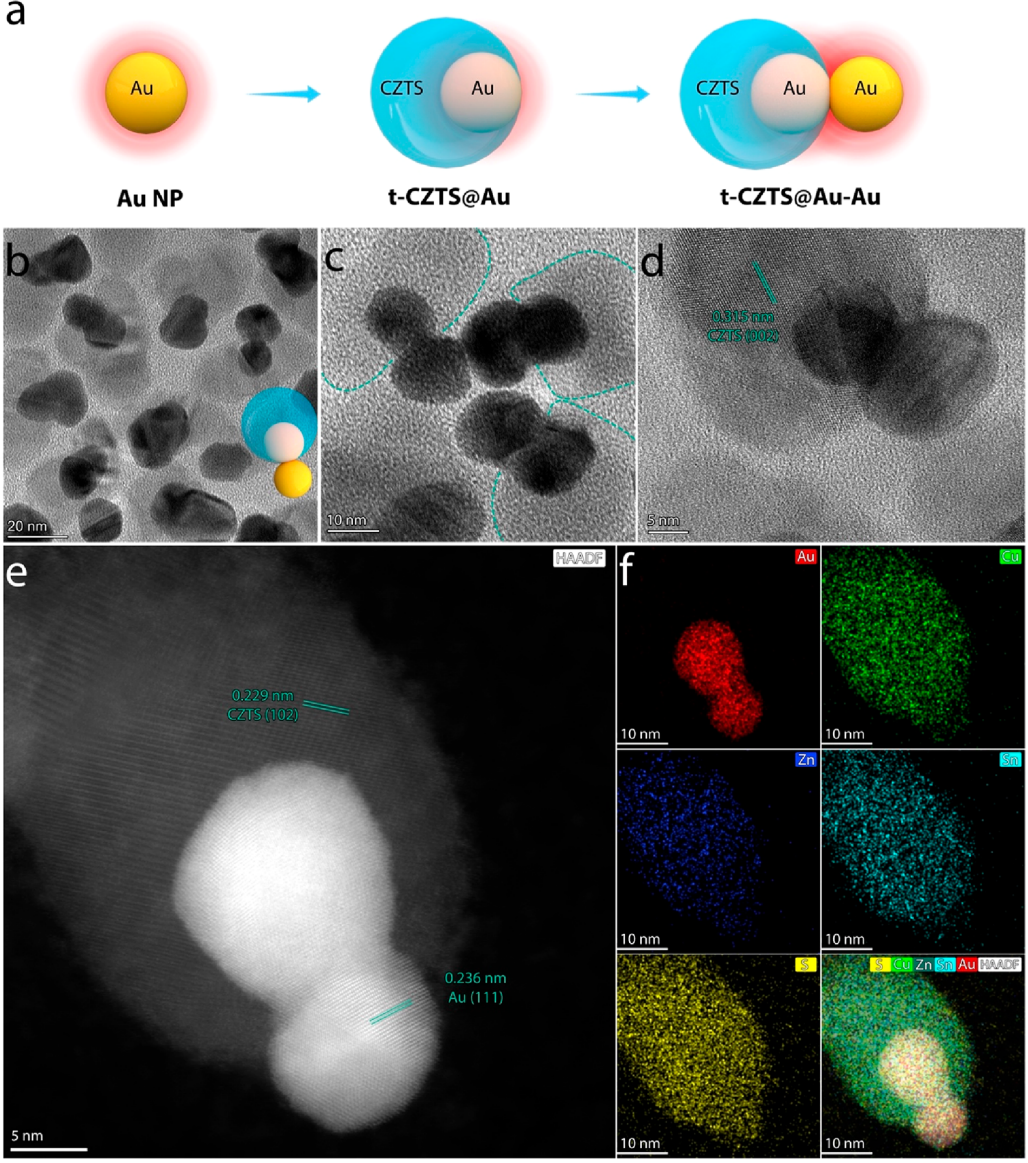
(a) Schematic illustration of the fabrication of t-CZTS@Au–Au HNSs. (b–d) TEM images of t-CZTS@Au–Au HNSs with different scales of magnification. The dot lines in (c) denote the perimeters of CZTS. (e) High resolution HAADF-STEM image and (f) the corresponding elemental mappings of one t-CZTS@Au–Au HNS.

Having in mind that positioning the singularities of Au–Au dimers at CZTS holds the promise to enhance the photocatalytic activity, the challenge becomes the realization of such unprecedented heterostructures.^46−52^ In this work, we fabricated the t-CZTS@Au–Au HNSs by developing a colloidal strategy coupling templating with seed-mediated growth. As illustrated in Figure 1a, a presynthesized Au NP is first encapsulated in CZTS in a *tangent* geometry that the Au NP kisses the CZTS (the resulting structure denoted as **t-CZTS@Au**). With such a geometric configuration, the t-CZTS@Au presents a narrow, CZTS-noncovered Au surface (see Figure S2 for HR-(S)TEM) images) which acts as a seeding site for the subsequent growth of the second Au NP.^52^ From the synthetic perspective, the CZTS essentially plays the role of a physical template that breaks the symmetry of a Au NP during the seeded growth, leading to the formation of a Au NP-Au NP dimer. As a characteristic of such a geometry, the singularity of the Au NP-Au NP dimer where the LSPR-induced field enhancement is most intense is precisely architected at the surface of CZTS.

Figure 1b presents a typical TEM image of the realized t-CZTS@Au–Au HNSs. Based on the distinct contrasts between Au and CZTS, calabash-like Au–Au dimers of a dark color contrast are clearly distinguished from CZTS NPs having a much lighter contrast. The Au–Au dimers contain two discrete spherical NPs with the size of ∼18 nm, while the CZTS NPs exhibit an anisotropic shape with an average size of ∼40 nm. Of note, due to the random dispersion of the HNSs on the TEM grid, typically, part of the outer Au NP visually superimposes the inner Au NP and CZTS NP in the TEM image. By properly tilting the specimen, the realistic geometry of the HNSs, i.e., the outer Au NP being segregated from the CZTS NP (Figure 1c) and the whole Au entity being of a dimeric rather than spherical morphology (Figure S3) can be visualized. In accordance with this geometric feature, a representative HR-TEM image of one single t-CZTS@Au–Au HNS (Figure 1d) shows that the perimeter of the CZTS NP crosses through the sharp corner formed by the curved surfaces of the two kissing Au spheres. HAADF-STEM clearly confirms the presence of two different materials (i.e., Au appears brighter than CZTS) configured in the as-described geometry (Figure 1e, Figure S4). The corresponding EDX elemental mappings reveal the uniform distribution of the Au element in the higher-contrast Au–Au dimer and Cu, Zn, Sn, and S elements in the lower-contrast CZTS NP (Figures 1f, S4). Here, as determined by XPS, the Au element is in the metallic state (Au^0^), while Cu, Zn, Sn, and S elements exist in the form of Cu^I^, Zn^II^, Sn^IV^, and S^II^ species, respectively, agreeing well with the theoretical chemical states of CZTS (Figure S5). In addition, both the Au–Au dimers and the CZTS NPs are found to be highly crystalline. The measured lattice spacings of 0.315 and 0.229 nm can be respectively assigned to the (002) and (102) plane of wurtzite-phased CZTS, while 0.236 nm can be assigned to the (111) plane of cubic-phased Au. Consistent with these crystallographic insights from the TEM, the XRD pattern (Figure S6) exhibits two distinct sets of diffraction peaks arising from Wurtzite CZTS and face-centered-cubic Au, reiterating the formation of segregated CZTS and Au phase in the t-CZTS@Au–Au HNSs. Furthermore, from the combined mappings, it is clearly observed that one Au NP of the Au–Au dimer is embedded in the CZTS, while the other touches the CZTS from the outside. As such, the kissing point (plasmonic singularity) of the Au–Au dimer sits right at the surface of CZTS. Overall, the characterization results in Figure 1 and Figures S4–6 demonstrate the successful fabrication of an HNS (i.e., **t-CZTS@Au–Au**) that comprises CZTS embracing one Au NP of the Au–Au dimer.

To elucidate the mechanism of plasmonics-facilitated hydrogen generation, we fabricated another four Au/CZTS hybrid configurations (Figure 2). Apparently, it was not trivial to construct the exquisite **t-CZTS@Au–Au** HNSs. The relative position of the first Au NP with respect to the CZTS NP is crucial, as it determines the exposure area of the Au NP seeding the secondary growth of the other Au NP and thus the geometry of the resulting CZTS@Au–Au HNSs. Interestingly, the position control can be realized experimentally by regulating the amount ratio of the CZTS to the Au NP seeds (see Figure S7 for the mechanisms). Figure 2b shows the TEM image of t-CZTS@Au, which acts as the precursor with adequate seeding exposure for the growth of t-CZTS@Au–Au. When the first AuNP is *fully* embedded in CZTS, the resulting hybrid nanostructure, **f-CZTS@Au** (Figure 2a), does not present any exposed Au surface for the formation of conjoined Au–Au dimers. On the other hand, if the first Au NP is only *partially* enclosed in CZTS, the resulting hybrid nanostructure, **p-CZTS@Au** (Figure 2c), presents a larger exposed area and hence induces less anisotropic growth of Au, yielding a new trimeric nanostructure (denoted as **p-CZTS@Au–Au**, Figure 2d) where the initial Au NP evolves into an elongated entity rather than into two discrete domains as observed in t-CZTS@Au–Au. Obviously, the degree of singularity is much less remarkable in p-CZTS@Au–Au than in t-CZTS@Au–Au (see the HR-S(TEM) images in Figure S8 for the pronounced difference). As control samples for studying the enhancement mechanism of photocatalysis, the four Au/CZTS HNSs (i.e., f-CZTS@Au, t-CZTS@Au, p-CZTS@Au, p-CZTS@Au–Au) as well as pure Au NPs and pure CZTS NPs were all produced and characterized (Figure 2 and Figures S9–S14).

**Figure 2:**
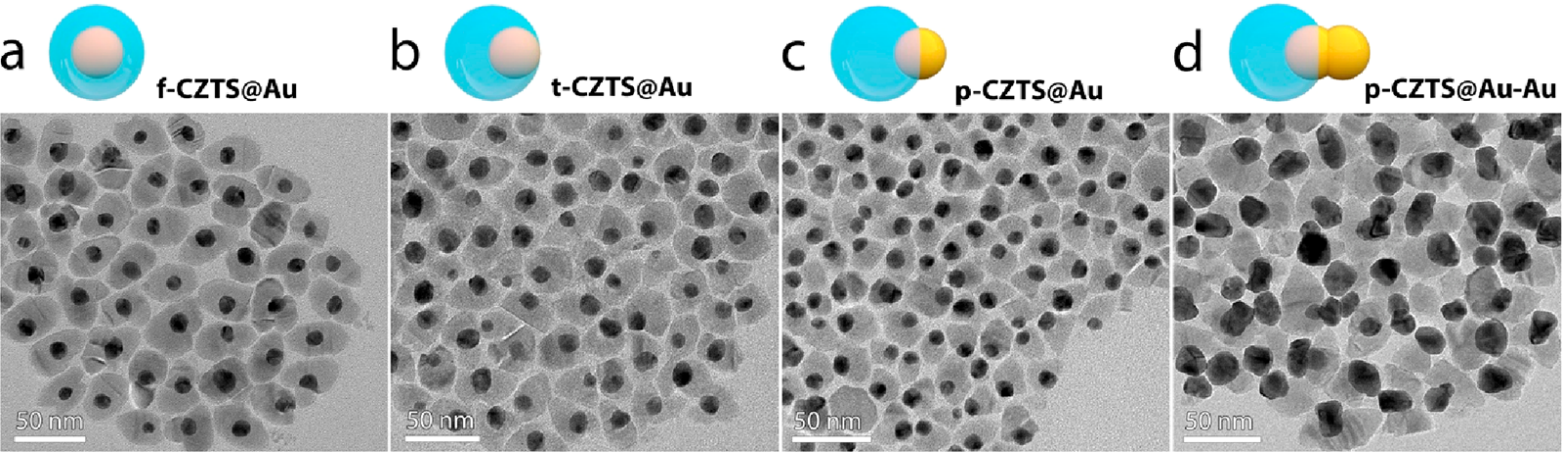
Scheme and corresponding TEM image of Au/CZTS hybrid configurations including (a) f-CZTS@Au, (b) t-CZTS@Au, (c) p-CZTS@Au, and (d) p-CZTS@Au–Au.

The distinct structural characteristics of these HNSs are reflected in their optical properties. As shown in the UV–vis–NIR spectra (Figure 3a), the CZTS NPs exhibit a broad absorption extending to near-infrared (∼825 nm), while the Au NPs possess a prominent plasmonic peak at ∼526 nm. The f-CZTS@Au resembles CZTS in the broad absorption, but is further characterized by a new Au plasmonic peak at ∼ 650 nm, which is much weakened, broadened, and red-shifted, as compared to that of the bare Au NPs. This pronounced change in the absorption property of the Au NPs can be jointly attributed to the delocalization of the Au plasmon over the semiconductor CZTS and the change in the refractive index of the medium surrounding the Au NPs from chloroform (∼1.4) to CZTS (∼2.5).^53,54^ Moreover, the thick opaque CZTS-shell (∼17 nm, Figure 2a) shielding the light from interacting with the inner Au NPs should also contribute to the drastic weakening of the Au plasmonic absorption. As the Au NP is increasingly outward from the CZTS and thus more accessible for light interaction, the absorption band of the hybrid nanostructure (i.e., t/p-CZTS@Au) moves more toward that of the bare Au NPs. Regardless of the different light responses, the three f/t/p-CZTS@Au HNSs all demonstrate that the incorporation of the Au NP into CZTS results in hybrid nanostructures with markedly enhanced optical absorption in the visible range compared with the pure CZTS. With an additional Au NP attached, the t-CZTS@Au–Au manifests an even broader and intensified absorption between 500 and 1000 nm. Such intriguing optical properties are not simply due to the addition of plasmonically active Au, but unique to the geometric structure (i.e., dimeric structure with a singularity). p-CZTS@Au–Au, the HNS with Au added but more to coalesce the initial Au NP than to form a separate domain yielding a singularity, has a broad but lower absorption. While the size/morphology distribution can contribute (see the theoretical analysis later), the broadband absorption of the t/p-CZTS@Au–Au originates from the plasmonic singularity and the resonance of the elongated Au entity.^37,55^ Overall, these results demonstrate the striking effect of nanostructures’ geometry on their optical properties, which can in turn affect their photocatalytic activities.

**Figure 3:**
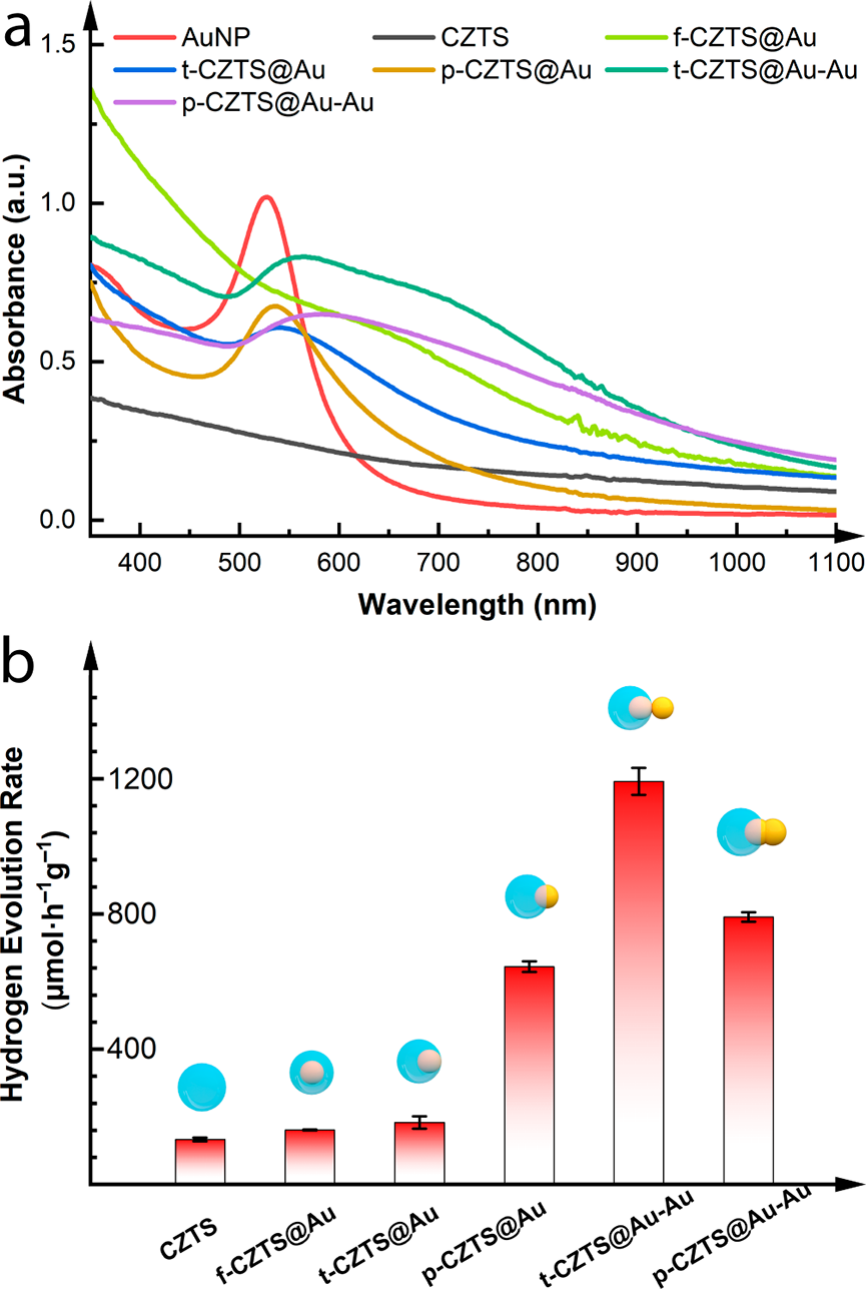
(a) Experimental absorption spectra of various Au/CZTS HNSs in comparison with pure Au NPs and pure CZTS NPs. The concentration of Au element in all Au-containing samples is 0.05 mg mL^–1^ and the concentration of CZTS in the CZTS sample is 0.02 mg mL^–1^. (b) Photocatalytic hydrogen production rates normalized by the full mass of the Au/CZTS photocatalysts. The rates normalized by only the mass of CZTS are also reported in Figure S15 that shows a similar activity trend as presented here.

The photocatalytic hydrogen evolution activity of t-CZTS@Au–Au was then measured and compared with those of other samples mentioned above (Figure 3b). The pure CZTS NPs show a low hydrogen production rate (HPR, 133 μmol h^–1^ g^–1^) owing to its poor light-harvesting ability and rapid recombination of photogenerated charge carriers.^43^ All other HNSs with the incorporation of Au NP boost the hydrogen yield to different extents. For f/t/p-CZTS@Au HNSs, as the Au NP locates further away from the center of the CZTS component, the HPR increases more significantly. In particular, p-CZTS@Au with the Au NP largely uncovered exhibits the HPR (644 μmol h^–1^ g^–1^) 4.8-fold higher than that of the CZTS. Since it was challenging to further move the Au NP out of the CZTS from the synthetic perspective, p-CZTS@Au–Au featuring more Au exposed was prepared, which delivers an even higher activity of 791 μmol h^–1^ g^–1^. Interestingly, despite less Au being exposed, t-CZTS@Au–Au performs yet better than p-CZTS@Au–Au, or in other words, the best among all the tested HNSs, with the HPR up to 1192 μmol h^–1^ g^–1^, corresponding to a 9-time enhancement over that of CZTS. After five consecutive cycles of 6 h of reaction, t-CZTS@Au–Au largely retains the activity, demonstrating its good stability (Figure S16). To the best of our knowledge, such a superior activity of t-CZTS@Au–Au surpasses the performance of all the reported plasmonic Au/Ag/CZTS HNSs and is even comparable with the state-of-the-art CZTS/Pt HNSs that consist of the eminent H_2_-evolution catalyst Pt (Figure S1). By further employing photoelectrochemistry, we verified that the LSPR promoted the photoelectrons over a broad spectral range for chemical reactions (see Figures S17–19 and discussion).

The prominent increment of photocatalytic activity by geometric design at the nanoscale is interesting and appealing for other related chemical reactions. However, a comparison of absorption spectra among HNSs cannot adequately explain their disparities in the improvement of hydrogen production. Particularly, p/t-CZTS@Au–Au share a similar ability to capture photons while varying their catalytic activities significantly (Figure 3). Consequently, we implemented FDTD simulations for a better understating of the mechanism behind the photocatalytic process. We simulated all the six configurations used for hydrogen evolution, with the calculated optical responses summarized in Figure 4. By comparing f-CZTS@Au with CZTS (Figure 4a, b), it is obvious that the introduction of the first Au NP in contact with CZTS perturbs the absorption of the CZTS NP (the black dashed line). Additional photons around the plasmonic resonance of Au NP are captured by CZTS due to the near-field enhancement. Despite the volume decrement, the averaged absorption in the visible is enhanced in the CZTS NPs (Figure 4g). Meanwhile, the introduced AuNPs themselves assist the hybrid system to capture more photons. However, the improvement of absorption, or the generation of carriers, does not necessarily enhance hydrogen generation. The carrier produced in the center of f-CZTS@Au might not be able to reach the surface for chemical reactions, due to the requirement of charge neutrality. Correspondingly, field enhancement in CZTS induced by the plasmonic effect from the Au NP may be the primary factor for additional carriers consumed in the hydrogen evolution. These facts explain the limited enhancement of the photocatalytic activity delivered by f-CZTS@Au. As we move the Au NPs away from the center, the averaged absorption does not experience a substantial change in f/t/p-CZTS@Au (Figure 4g). However, conspicuous field enhancement around Au is observed, accompanied by regions with enlarged absorption power close to the CZTS-liquid interface (Figure 4c, d), which can be the driving force for the improvement of the catalytic activity shown in Figure 3b. Both the absorption and field can be further enhanced by the introduction of the second Au NPs, as shown for t/p-CZTS@Au–Au (Figure 4e, f). The observed similar averaged absorption (strictly, p-CZTS@Au–Au even absorbs slightly more than t-CZTS@Au–Au) yet different catalytic abilities (t-CZTS@Au–Au performs 1.5-fold better than p-CZTS@Au–Au) very likely originates from the hot-electron driven photocatalysis, though electromagnetic field excitation and plasmon-induced resonance energy transfer might also contribute.^24,56,57^ The plasmonic hotspot between the two Au NPs serves as a source of energy carriers for photocatalysis (Figure 4e, f). The brightest hotspot of t-CZTS@Au–Au is exactly located at the interface among Au, CZTS and liquid, facilitating the transport of the hot electrons to the site for chemical reactions. On the contrary, a large portion of the hot electrons produced in p-CZTS@Au–Au is comparatively far away from the CZTS/liquid interface, dramatically reducing the effective usage of the hot carriers. Thus, these results demonstrate that the photocatalytic activity is closely related with the geometric structure, in addition to the optical absorption, of the photocatalysts. Considering the bluntness of the contact and the size fluctuations in realistic samples, we further implemented an investigation into the transformation optics for realistic configurations (Figures S21 and 22). The unique feature of adiabatic focusing is preserved in spite of the bluntness, driving photons at different wavelengths to concentrate and synergically facilitate the generation of hot electrons for the chemical reaction.

**Figure 4:**
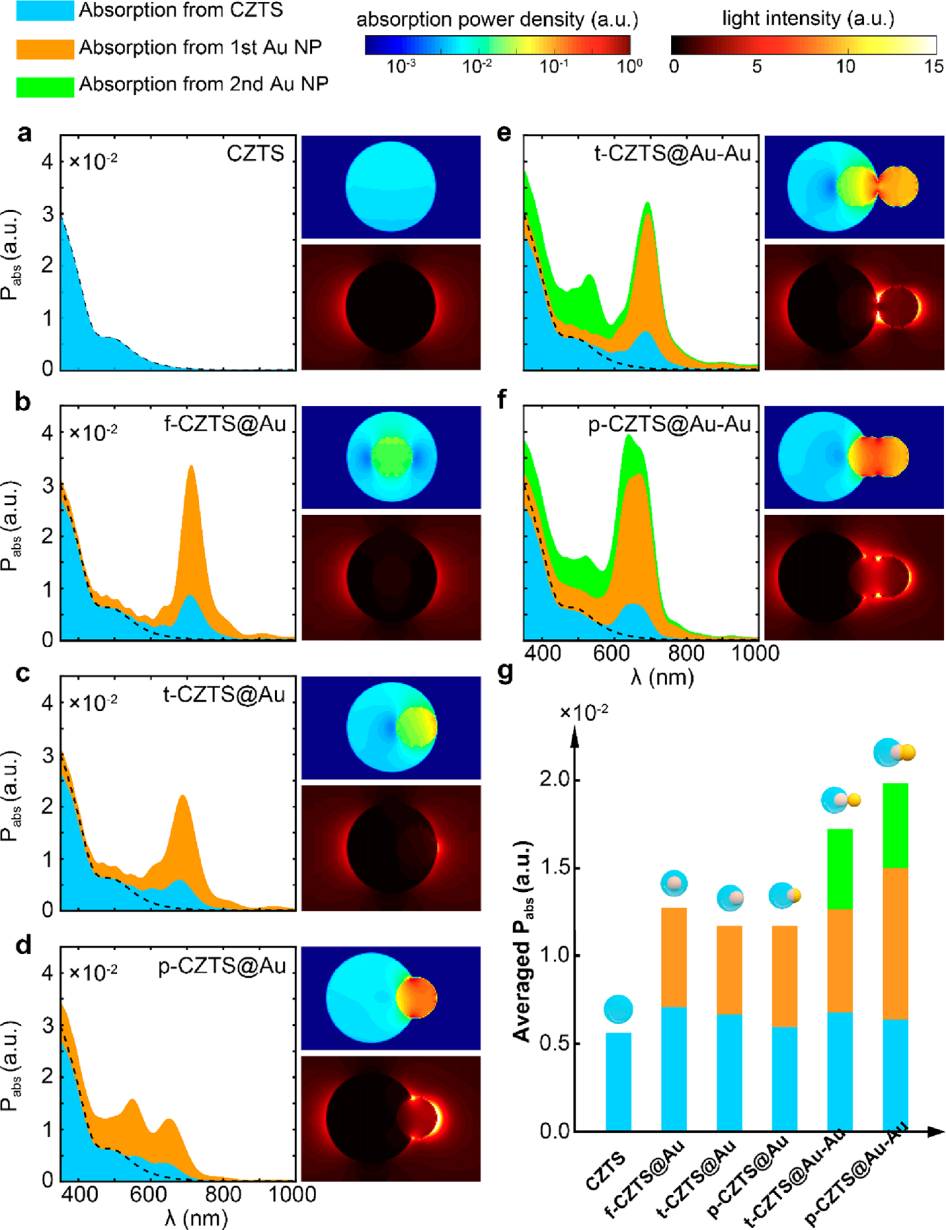
FDTD simulations of light absorption and near-field enhancement of (a) CZTS, (b) f-CZTS@Au, (c) t-CZTS@Au, (d) p-CZTS@Au, (e) t-CZTS@Au–Au, and (f) p-CZTS@Au–Au. For each configuration, the left panel shows the power absorption spectrum from 350 to 1000 nm obtained by averaging the light absorption along the three orthogonal polarization directions of the incident light. Absorption from different components of each HNS is differentiated in different colors (i.e., blue, orange, and green for CZTS, first Au NP, and second Au NP, respectively). The top-right panel and the bottom-right panel illustrates the spatial distribution of absorption power density and light intensity at 532 nm, respectively. The polarization of the electric field is along the axial axis where the light-matter interaction is strongest. The spatial distribution under other two wavelengths (405 and 808 nm) is also provided in Figure S20, demonstrating a similar effect. (g) Absorption power for different HNSs averaged over the spectrum from 350 to 825 nm, wherein the CZTS can be excited. Black dashed line in a–f is a guide, showing the absorption spectrum of CZTS NP only.

To investigate the temporal dynamics of the hot electrons generated inside the nanoparticles, we implemented a set of measurements of transient absorption (Δ*A*) based on a pump–probe setup (Figure 5a).^58^ The negative absorption before delay time 1 ps corresponds to absorption saturation at the band edge states in semiconductors, while the positive transient values are due to accumulation of hot carriers near the band edges, which causes excited state absorption transitions into higher energy states. As the introduction of Au particles into CZTS host, the lifetime (τ) is improved from 0.30 to 0.48 ps. When Au NPs shift from the center, the lifetime is extended to 1.45 ps, agreeing well with near-field enhancement (Figure 4) and improved hydrogen evolution rate (Figure 3b). The lifetime is further increased to >3 ps (with 3.36 ps for p-CZTS@Au, 3.11 ps for p-CZTS@Au–Au and 3.29 ps for t-CZTS@Au–Au), resulting from gigantically enhanced light intensity at the Au/CZTS interface. Based on the match among numerical simulations (Figure 4), hydrogen evolution (Figure 3b) and transient absorption (Figure 5a), we may attribute the energetic carriers produced in Au near the triphase region to be the primary driving force for proton reduction (Figure 5b).

**Figure 5:**
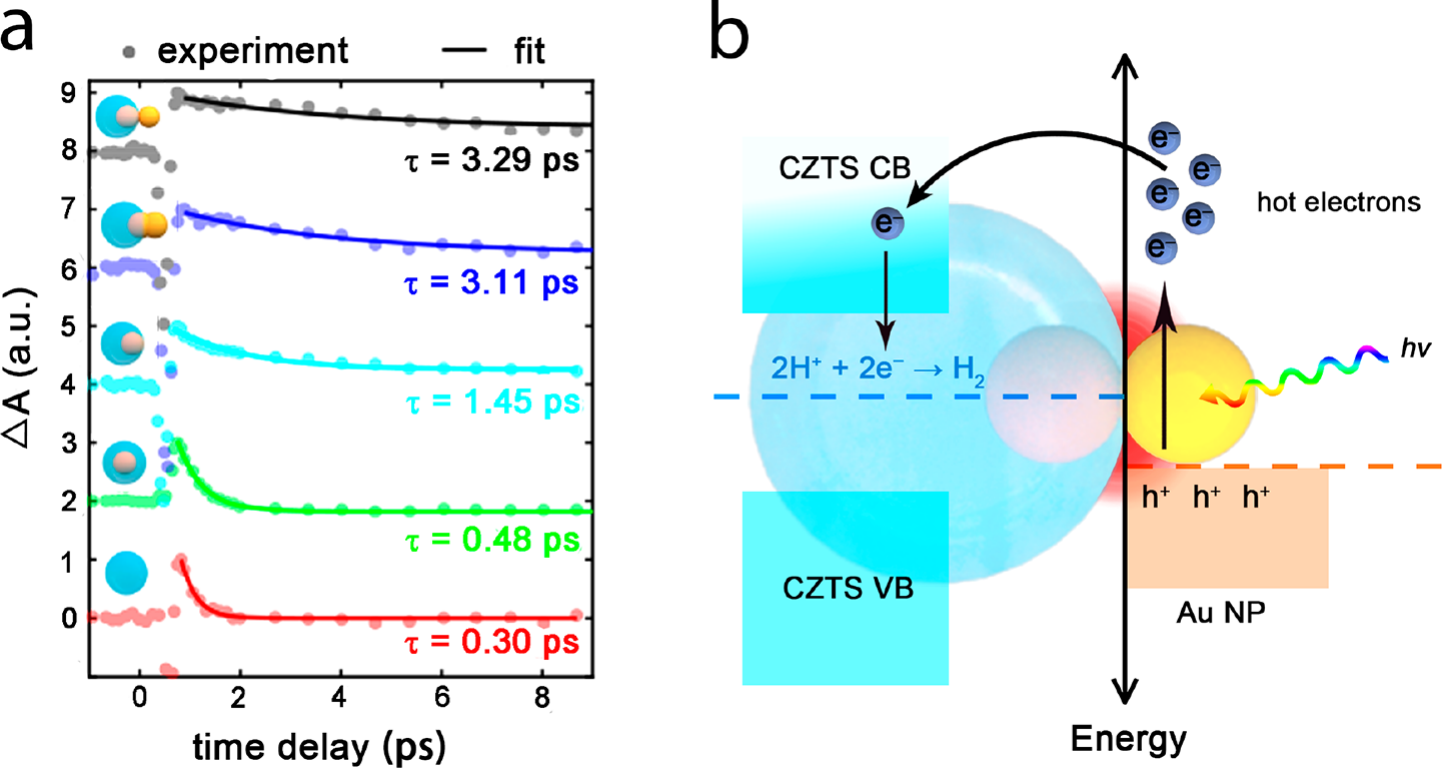
(a) Transient absorption response of various Au/CZTS HNSs and CZTS. A pulse laser of 650 nm at a fluence of 17.63 J/m^2^ operates as the pump, while the wavelength of the probe laser is 770 nm (1.6 eV), matching the transition going beyond the bandgap of CZTS (∼1.5 eV). The experimental data (circles) is fitted by an exponential decay (solid lines) to extract the lifetime (τ), marked on each curve, of fast-decaying modes. (b) Schematic illustration of interfacial charge transfer and photocatalytic hydrogen evolution at the singular region in t-CZTS@Au–Au HNS. Note that the photoexcitation of CZTS is not depicted in the schematic diagram, considering its relatively minor contribution to the hydrogen evolution.

In summary, we have developed a novel colloidal strategy by combining templating and seeded growth to achieve a transformation-optics-inspired light harvesting hybrid nanostructure comprising CZTS and Au–Au dimer (t-CZTS@Au–Au). A plasmonic singularity arising from the Au–Au dimer is precisely positioned at CZTS surface where photocatalytic H_2_ production occurs. Through verification with a series of synthetically controlled Au/CZTS hybrid nanostructures, we demonstrated that the broad light interaction and the strong near surface electromagnetic field, both endowed by the unique geometry, led to a significant photocatalytic performance enhancement (∼9 times), with the hydrogen evolution rate reaching 1192 μmol h^–1^ g^–1^. Combining numerical and experimental investigations, we verified that both the sharpness of the singular feature (or equivalently brightness of the hotspot) and the relative position to the reactive site play an important role in optimizing the photocatalytic activity. Although the quantum efficiency of the t-CZTS@Au–Au hybrid nanostructure needs improvement (see Figure S23 for discussion), the new concept of plasmonic photocatalysis–creating a plasmonic singularity and placing it to the reactive site where the photocatalytic reaction takes place, stimulates appealing strategies for developing composite plasmonic-metal/semiconductor photocatalysts that are manifesting themselves increasingly in green fields.^59^

## Supporting Information Available

Experimental details, results and discussion (chemicals and materials, synthetic procedures, characterizations, photoelectrochemical test, electrochemical impedance spectrometry, photocatalytic hydrogen evolution test, recycling experiment, FDTD simulation, pump–probe experiments, calculation of the apparent quantum efficiency, photoelectrochemistry results and discussion), and Figures S1–S23 (PDF)
