# Supplementary material for: Transformation-Optics-Designed Plasmonic Singularities for Efficient Photocatalytic Hydrogen Evolution at Metal/Semiconductor Interfaces
Source: Nano Lett. 2023 May 26;23(11):5288–96. doi: 10.1021/acs.nanolett.3c01287 (PMC10273458; doi:10.1021/acs.nanolett.3c01287)
Supplement: Supplementary file 1 — nl3c01287_si_001.pdf [file nl3c01287_si_001.pdf]

Supporting Information for

Transformation-Optics-Designed Plasmonic Singularities for Efficient Photocatalytic Hydrogen Evolution at Metal/Semiconductor Interfaces

Tingting Lin,[1] Tianyi Yang,[1] Yuhang Cai,[3] Jingwei Li,[1] Guangxiang Lu,[1] Shuangqun Chen,[1] Yi Li,[5] Liang Guo,[3] Stefan A. Maier,[2,6,7*] Changxu Liu[4,7,8*] and Jianfeng Huang[1*]*

[1] State Key Laboratory of Coal Mine Disaster Dynamics and Control, Institute of Advanced Interdisciplinary Studies, School of Chemistry and Chemical Engineering, Chongqing University, Chongqing 400044, China

[2] School of Physics and Astronomy, Monash University, Clayton, Victoria 3800, Australia

[3] Department of Mechanical and Energy Engineering, Southern University of Science and Technology, Shenzhen 518055, China

[4] Centre for Metamaterial Research & Innovation, Department of Engineering, University of Exeter, Exeter EX4 4QF, UK

[5] School of Microelectronics, MOE Engineering Research Center of Integrated Circuits for Next Generation Communications, Southern University of Science and Technology, Shenzhen 518055, China

[6] Blackett Laboratory, Imperial College London, London SW7 2BZ, United Kingdom

[7] Chair in Hybrid Nanosystems, Nanoinstitute Munich, Faculty of Physics, Ludwig Maximilians University of Munich, 80539 Munich, Germany

[8] Department of Mathematics, Physics and Electrical Engineering, Northumbria University, Newcastle Upon Tyne NE1 8ST, United Kingdom

E-mail: guol3@sustech.edu.cn, stefan.maier@monash.edu, c.c.liu@exeter.ac.uk, jianfeng.huang@cqu.edu.cn

1. Experimental Section

(1) Chemicals and Materials

Copper(II) acetate monohydrate ($Cu(OAc)_2 \cdot H_2O$, 99%), zinc acetate dehydrate ($Zn(OAc)_2 \cdot 2H_2O$, 99.99%), gold(III) chloride trihydrate ($HAuCl_4 \cdot 3H_2O$, ≥99.9%), oleylamine (80-90%) and 3-mercaptopropionic acid (98%) were purchased from Shanghai Aladdin Biochemical Technology Co., Ltd. Tin(II) acetate ($Sn(OAc)_2$, 95%), 1-dodecanethiol (1-DDT, 98%), tert-dodecanethiol (t-DDT, 98%) were bought from Shanghai Macklin Biochemical Technology Co., Ltd. Trichloromethane ($CHCl_3$, AR), toluene and ethanol were obtained from Chengdu Kelong Chemical Co., Ltd. All chemicals were used as received without further purification. All aqueous solutions were prepared using deionized (DI) water with a resistivity of 18.2 MΩ·cm.

(2) Sample Fabrication

Synthesis of 18 nm Au NPs: A $HAuCl_4$ solution (10 mM) was first prepared by dissolving $HAuCl_4 \cdot 3H_2O$ powders in oleylamine. To synthesize the 18 nm Au NPs, the $HAuCl_4$ solution (625 μL, 10 mM) was stirred at 120 °C for 12 min and then half of the resulting solution was replaced with pure oleylamine (312.5 μL), followed with another heating at 85 °C for 2h. Four aliquots of $HAuCl_4$ (each containing 625 μL, 10 mM) were then repetitively added into the above reaction solution every hour. After that, the reaction solution was cooled down naturally and then washed and redispersed either in $CHCl_3$ for absorption test or in oleylamine for seeding growth of CZTS/Au HNSs.

Synthesis of Wurtzite CZTS NPs: Wurtzite CZTS NPs were prepared following the reported method with modifications.[1] To be specific, $Cu(OAc)_2 \cdot H_2O$ (32.0 mg), $Zn(OAc)_2 \cdot 2H_2O$ (22 mg), $Sn(OAc)_2$ (23 mg) and oleylamine (4 mL) were mixed in a three-necked flask and degassed for 2 hours at room temperature. After that, the temperature was raised to and maintained at 120 °C under N_2 bubbling for 30 min. A mixture of 1-DDT (0.05 mL) and t-DDT (0.35 mL) was subsequently injected, followed with a further increase of the temperature to 280 °C. After 30 min, the product formed and washed by centrifugation three times with ethanol and $CHCl_3$.

Synthesis of f-CZTS@Au, t-CZTS@Au and p-CZTS@Au: To synthesize f-CZTS@Au, the oleylamine used was all degassed under vacuum for at least 30 min and then stored in N_2. $Cu(OAc)_2·H_2O$ (14.0 mg), $Zn(OAc)_2·2H_2O$ (9.6 mg), $Sn(OAc)_2$ (10.1 mg) and oleylamine (1.75 mL) were mixed in a two-necked flask and degassed for 2 hours at room temperature, followed with heating at 120 °C under N_2 bubbling for another 30 min. The previously synthesized Au NPs were then quickly injected into the reaction flask, after which the reaction solution was stirred at 120 °C for another 2 min, before the injection of a mixture of 1-DDT (22 µL) and t-DDT (153 µL). Subsequently, the temperature was raised to and maintained at 280 °C for 30 min. After that, the reaction solution was cooled down naturally. The product was washed three time by centrifugation with ethanol and $CHCl_3$. Finally, the product was redispersed in $CHCl_3$ and stored in dark in a refrigerator for further use. **t-CZTS@Au** and **p-CZTS@Au** were synthesized using the similar procedures, with the exception that different amounts of reagents were used. Specifically, 12.8 and 8.0 mg $Cu(OAc)_2·H_2O$, 8.8 and 5.5 mg $Zn(OAc)_2·2H_2O$, 9.2 and 5.8 mg $Sn(OAc)_2$, 1.45 and 1.0 mL oleylamine, 15.0 and 12.5 µL 1-DDT, 105.0 and 87.5 µL t-DDT were used for t-CZTS@Au and p-CZTS@Au, respectively. The obtained t-CZTS@Au and p-CZTS@Au were finally either redispersed in $CHCl_3$ and stored in dark in a refrigerator for further use or redispersed in 4.5 mL oleylamine for seeding growth of t-CZTS@Au-Au and p-CZTS@Au-Au, respectively.

Synthesis of t-CZTS@Au-Au and p-CZTS@Au-Au: t-CZTS@Au was used as the seed for the preparation of t-CZTS@Au-Au. Specifically, 1.1 mL of the above t-CZTS@Au, $HAuCl_4$ (10 mM, 1.0 mL) and oleylamine (20 mL) were first mixed in a two-necked flask. The temperature was then raised to and kept at 120 °C for 30 min in dark under N_2 bubbling. The product was washed by centrifugation with ethanol and $CHCl_3$, and then re-dispersed in $CHCl_3$. p-CZTS@Au-Au was synthesized following the similar procedures, except that the seed was p-CZTS@Au instead.

(3) Characterizations

Electron Microscopy: Transmission electron microscopy (TEM), high-resolution TEM (HRTEM), energy dispersive X-ray (EDX) elemental maps and high-angle-annular-dark-field scanning TEM (HAADF-STEM) were performed on Talos 200 S/TEM (Thermo Fisher Scientific) operated at 200 kV or on Spectra 300 S/TEM (Thermo Fisher Scientific) operated at 300 kV.

X-ray Diffractometry (XRD): XRD was conducted on a PANalytical X'Pert Powder Advance instrument with Cu Kα radiation.

X-ray Photoelectron Spectroscopy (XPS): XPS data were collected on a ESCALAB 250Xi with a monochromatic Al K_α X-ray source under ultrahigh vacuum condition.

Inductively Coupled Plasma-Optical Emission Spectrometry (ICP-OES): ICP-OES was carried out on a Spectro GREEN model.

UV-vis-NIR Spectroscopy: The UV-vis-NIR absorption spectra in the range of 350-1000 nm were recorded on Shimadzu UV/Vis/NIR Spectrometer UV-3600.

(4) Photoelectrochemical Test

Photoelectrochemical tests were conducted in an acetonitrile solution containing 0.1 M ethyl viologen dipercholorate ($EV(ClO_4)_2$) and 0.1 M tetrabutylamonium hexafluorophosphate ($TBAPF_6$) at –0.9 V under Xe and laser illumination. Fluorine doped tin oxide (FTO) loaded with 1.0 mg catalyst served as the working electrode, while Pt and Ag/AgCl were used as the counter electrode and reference electrode, respectively. Working electrodes were prepared by evaporating the catalysts dispersed in $CHCl_3$. 300 W Xe lamp (light intensity: ~117 mW/cm^2, measured by a power meter) or 350 mW 405/532/808 nm lasers were used as the light source. The backgrounds of the dark currents in the tests were typically removed using the 'Baseline correction' function (Type: Polynom) of the 'LabSpec' software.

(5) Electrochemical Impedance Spectrometry (EIS)

EIS was conducted in the electrolyte containing 0.35 M Na_2S and 0.25 M Na_2SO_3 in the frequency range of 100 mHz−1 kHz with an ac signal of amplitude ∼10 mV at open bias.

(6) Photocatalytic Hydrogen Evolution Test

To better disperse the nanoparticles in the aqueous reaction solution, the original capping ligand oleylamine was replaced with 3-mercaptopropionic acid using a reported ligand exchange method.[2] Briefly, the concentrated solution of nanoparticles in $CHCl_3$ was mixed with 1 vol.% of 3-mercaptopropionic acid in formamide, followed with vigorous shake to promote the phase transfer in the bi-phase system. After that, the nanoparticles moved into the upper formamide phase, while the clear, colorless $CHCl_3$ solution was discarded. The nanoparticles in the formamide phase

were further purified with fresh $CHCl_3$ two times. Finally, the nanoparticles were precipitated with acetone and redispersed in DI water. The photocatalytic hydrogen evolution experiments were conducted in a 100 mL flask with stirring at 8 °C under the illumination of a 300 W Xe lamp equipped with an AM1.5G filter (light intensity: ~97 mW/cm^2, measured by a power meter) for 2 hours. Typically, 5 mg of nanoparticles, determined with the aid of ICP-OES, were dispersed in 50 mL deionized water containing 0.35 M Na_2S and 0.25 M Na_2SO_3 as hole scavengers. Before irradiating the reaction solution, the reactor was thoroughly purged with N_2 to remove all oxygen in the headspace of the reactor and dissolved in water. Each sample was tested for at least three times and the product hydrogen was analyzed by GC7900 system.

(7) Recycling Experiment

The recycling stability of the best-performing catalyst, t-CZTS@Au-Au, was evaluated. A total number of 5 cycles, each lasting 6 hours' illumination, was performed. The hydrogen was collected and quantified in each cycle. After each cycle, all the gas was discarded and the reactor was thoroughly re-purged with N_2. At the end of the fourth cycle, 10% of the hole scavengers was supplemented to examine its influence on the recycling stability.

(8) Finite Different in Time Domain (FDTD) Simulation

We performed numerical simulations using a FDTD Method. All the FDTD simulations were conducted with a commercial software (LUMERICAL, FDTD Solution). The refractive index of Au was adopted from ref.[3]. The refractive index of the background was set as 1.44, the refractive index of chloroform.[4] The refractive index of CZTS was adopted from ref.[5]. We set the size of the Au nanoparticles as 18 nm and the size of the CZTS nanoparticle as 40 nm, based on the statistical analysis of the TEM images of the corresponding samples. The absorption power density was calculated from $P_{abs} = -(1/2)\ \mathrm{Re}(\mathbf{E}\times\mathbf{H})$, wherein **E** is the electric field and **H** is the magnetic field.

(9) Transient Absorption/Pump-probe Experimental Details

Transient absorption pump-probe spectroscopy was based on a femtosecond laser with a central wavelength of 1030 nm, and a repetition rate of 5 kHz (Pharos 10 W, Light Conversion). The 1030 nm-output laser was split into two beams with a beam-splitter. One of the two beams

went through an optical parameter amplifier (Orpheus-F, Light Conversion) and was utilized as the probe with a pulse duration <200 fs. The other beam went through another optical parameter amplifier (Orpheus-N-2H, Light Conversion) and was utilized as the pump with a pulse duration <150 fs. The pump pulses were chopped at 500 Hz by a synchronized chopper, and the transient absorption signal was processed by a lock-in amplifier (SR860, Stanford Research System) to suppress the noise. The delay time between the pump and probe was controlled by a moving stage. The spot of the pump was elliptical, and the average spot diameter ($1/e^2$) was 170 μm. The spot diameter ($1/e^2$) of the probe was 50 μm.

(10) Calculation of the Apparent Quantum Efficiency (AQE)

The AQE of each CZTS-based photocatalyst studied in this work was calculated using the following equation:

$$\mathrm{AQE} = \frac{\text{Number of reacted electrons}}{\text{Number of incident photons}} = \frac{\text{Number of evolved H}_2\text{ molecules} \times 2}{\text{Number of incident photons}}$$

$\mathrm{AQE}_{t\text{-}CZTS@Au\text{-}Au} = 0.044\%$

$\mathrm{AQE}_{p\text{-}CZTS@Au\text{-}Au} = 0.028\%$

$\mathrm{AQE}_{p\text{-}CZTS@Au} = 0.025\%$

$\mathrm{AQE}_{t\text{-}CZTS@Au} = 0.0069\%$

$\mathrm{AQE}_{f\text{-}CZTS@Au} = 0.006\%$

$\mathrm{AQE}_{CZTS} = 0.005\%$

2. Supporting Figures

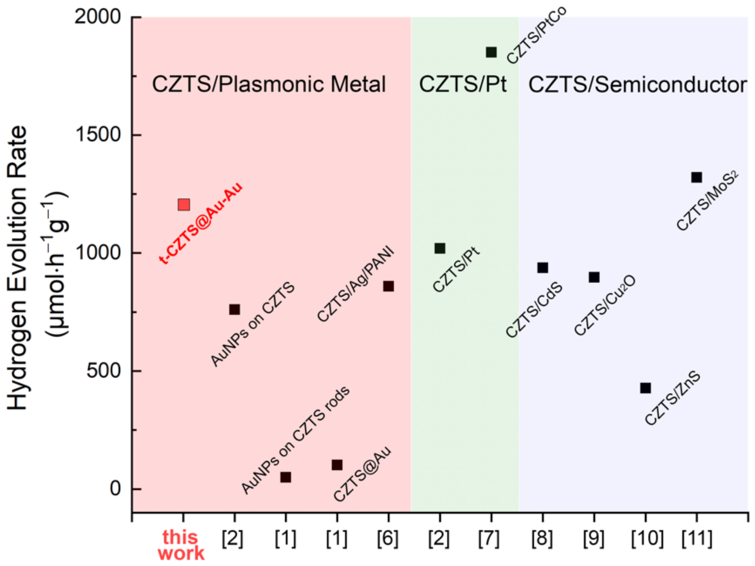

Figure S1. Comparison of hydrogen evolution rate between t-CZTS@Au-Au and other CZTS-based hybrid materials (including CZTS/Plasmonic Metal hybrids, CZTS/Pt Hybrids and CZTS/Semiconductor Hybrids) from the literature. [1, 2, 6-11]

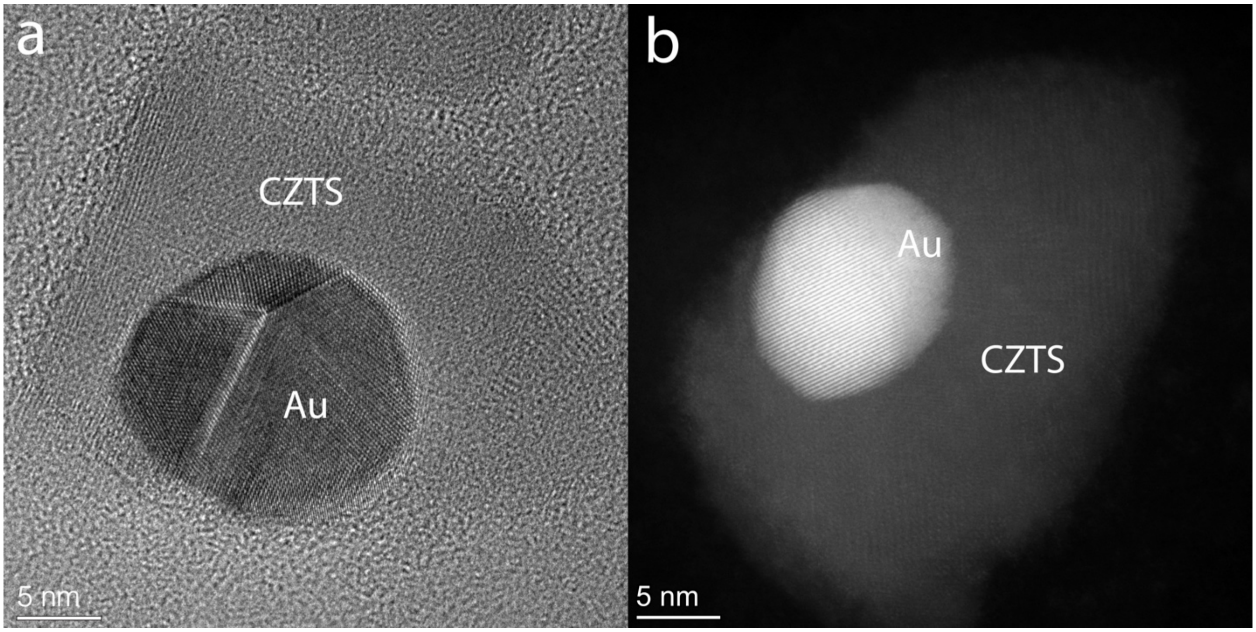

Figure S2. Aberration-corrected (a) HR-TEM and (b) HR-STEM image of a single t-CZTS@Au nanoparticle, showing a narrow, CZTS-noncovered Au surface.

Note: A large number of metallic dimers have been reported in the literature by manipulating the defects of the seeds, the growth kinetics, interfacial strains or using physical templates.[12-18] A straightforward approach to creating the t-CZTS@Au-Au HNSs hence could be controlled coating of one AuNP of the Au-Au dimers with CZTS. Nevertheless, the precise region-selective deposition of semiconductor CZTS on symmetric metallic dimers is challenging as well, because the distinct crystalline structures between CZTS and Au tend to drive complicated multiple island growth of CZTS on Au.[15]

Here, because Au and CZTS are of different chemical natures and the affinity of Au/Au is obviously higher than that of Au/CZTS, when t-CZTS@Au is exposed to the growth solution containing $HAuCl_4$ (Au precursor) and oleylamine (reducing agent), the newly formed Au atoms would preferentially deposit on the partially open surface of the Au NP rather than the surface of the CZTS, inducing the nucleation and overgrowth of the second Au NP.[18]

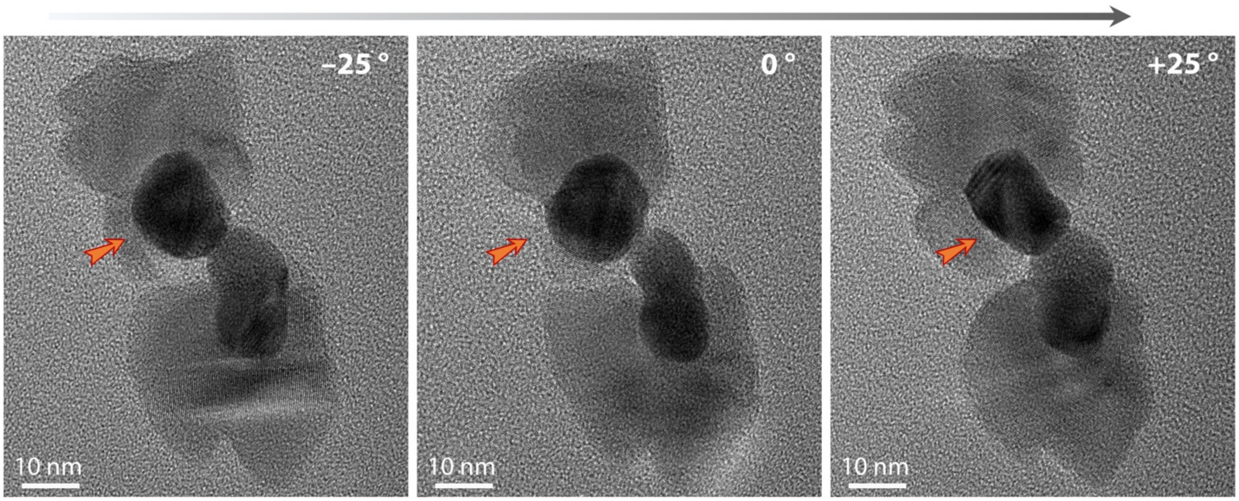

Figure S3. HRTEM images of t-CZTS@Au-Au NPs taken along three orientations, achieved by tilting the same specimen by 25° for each step. The arrows denote that one seemingly spherical Au NP progressively turns into a Au-Au dimer with the specimen tilting.

Note: Due to the random dispersion of the HNSs on the TEM grid, the axis connecting the centers of the two Au NPs and the CZTS NP is not always perpendicular to the TEM beam direction. As a consequence, typically, part of the outer Au NP visually superimposes the inner Au NP and CZTS NP in the TEM image. By properly tilting the specimen, the image, taken when the beam is normal to the axis, can then reflect the realistic geometry of the HNSs, i.e., the outer Au NP being segregated from the CZTS NP (**Figure 1c**) and the whole Au entity being of a dimeric rather than spherical morphology (**Figure S3**).

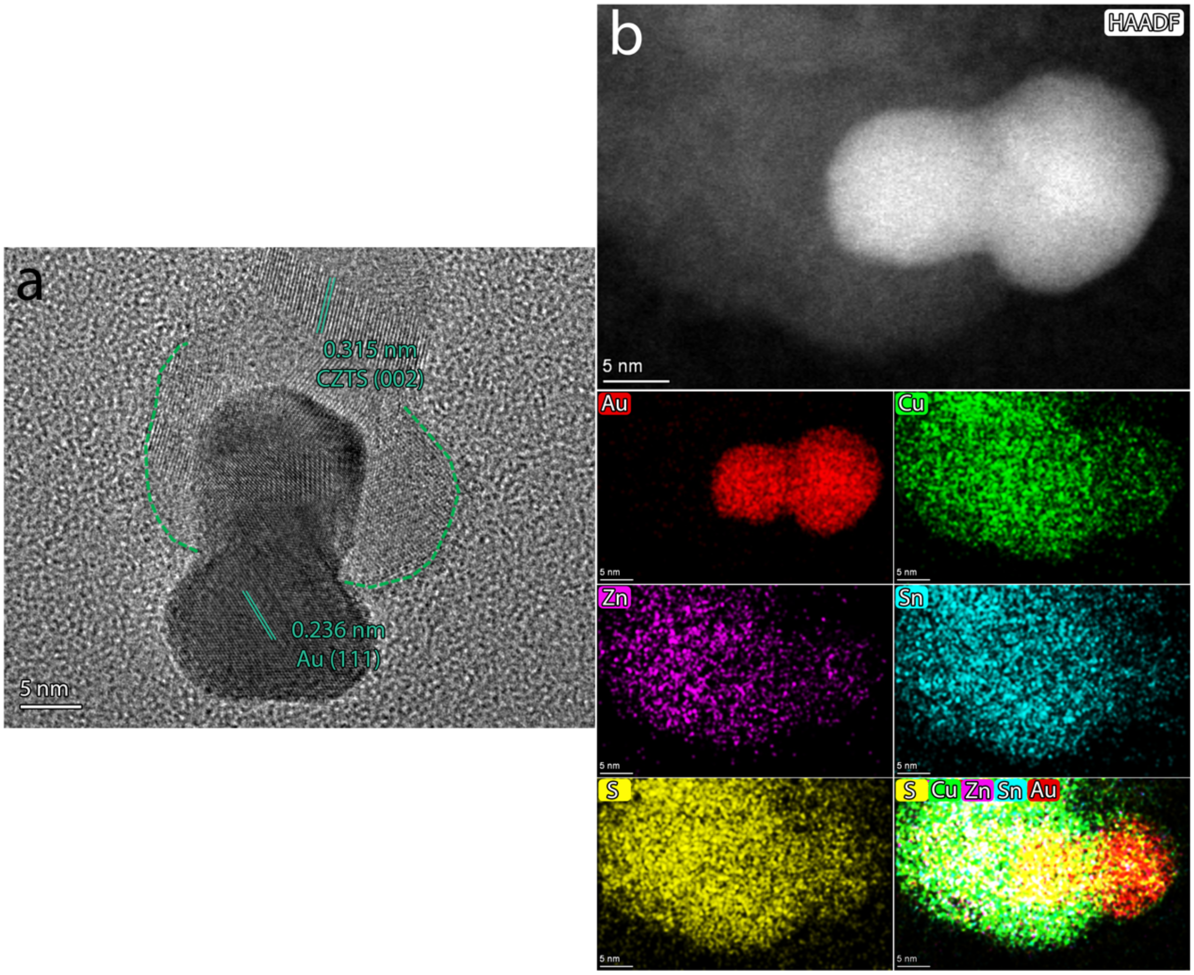

Figure S4. (a) HRTEM image and (b) HAADF-STEM image and the corresponding elemental mappings (bottom row) of t-CZTS@Au-Au HNSs. The dot lines in (a) denote the perimeters of CZTS.

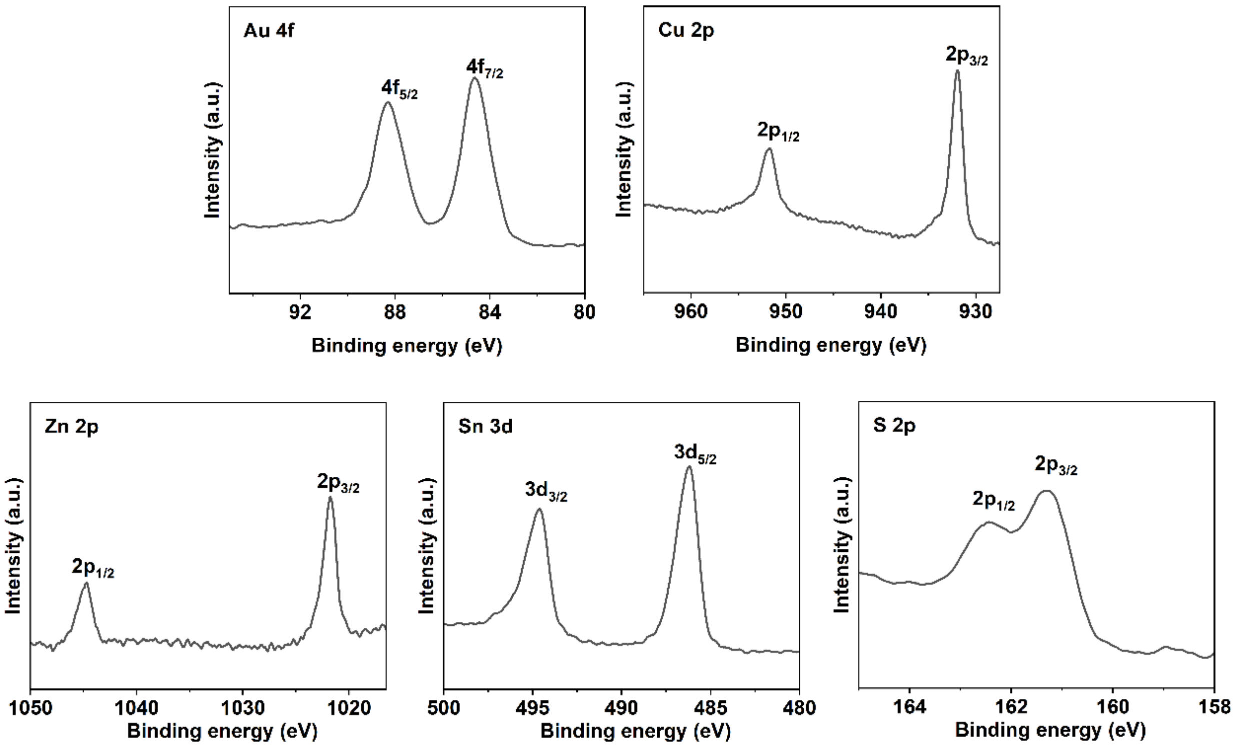

Figure S5. High-resolution XPS spectra of t-CZTS@Au-Au HNSs, showing the Au, Cu, Zn, Sn and S elements are present in the chemical state of Au^{0}, Cu^{I}, Zn^{II}, Sn^{IV} and S^{II}, respectively.

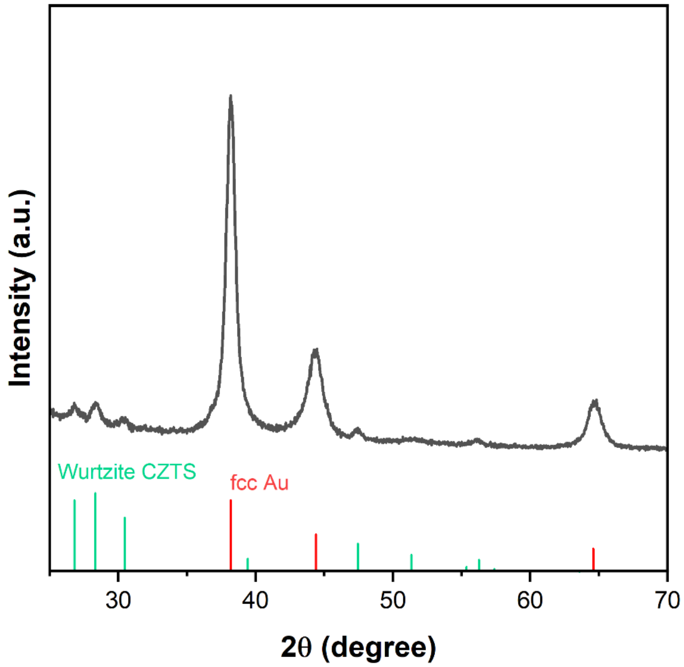

Figure S6. XRD pattern of t-CZTS@Au-Au HNSs.

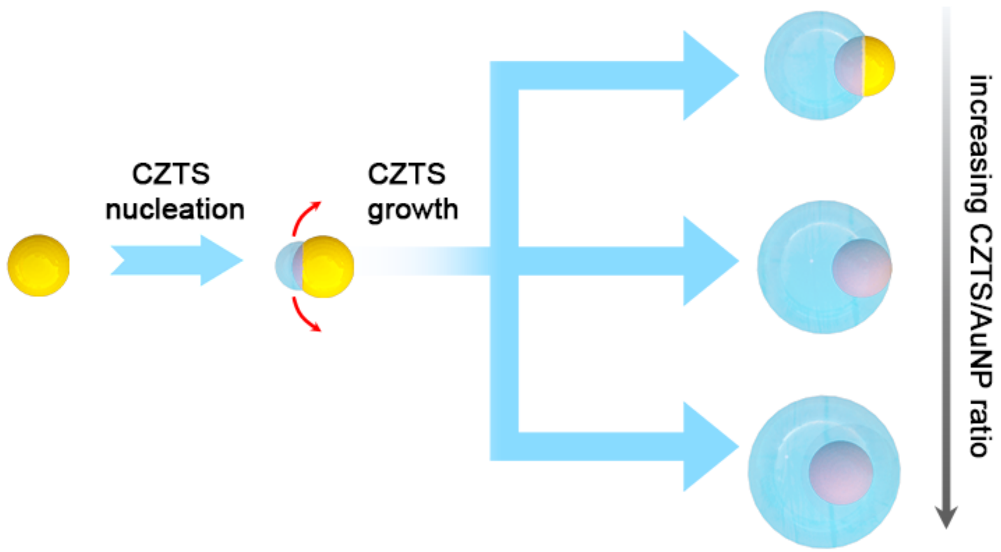

Figure S7. Schematic illustration of the synthetic mechanisms for f/t/p-CZTS@Au.

Note: In this work, CZTS@Au HNSs are synthesized by forming CZTS from the CZTS precursors on Au NPs. Specifically, CZTS nucleates on the surface of the Au NP and then grows under the provision of CZTS precursors. As more CZTS precursors are available, the CZTS grows bigger and forms a larger interfacial contact with the Au NP. Therefore, the f/t/p-CZTS@Au, with different degrees of CZTS encapsulation of the Au NP, can be synthesized by adjusting the amount of the CZTS precursors with respect to the amount of the Au NP seeds. Specifically, the p-, t- and f-CZTS@Au HNSs require an increasing amount of CZTS precursors.

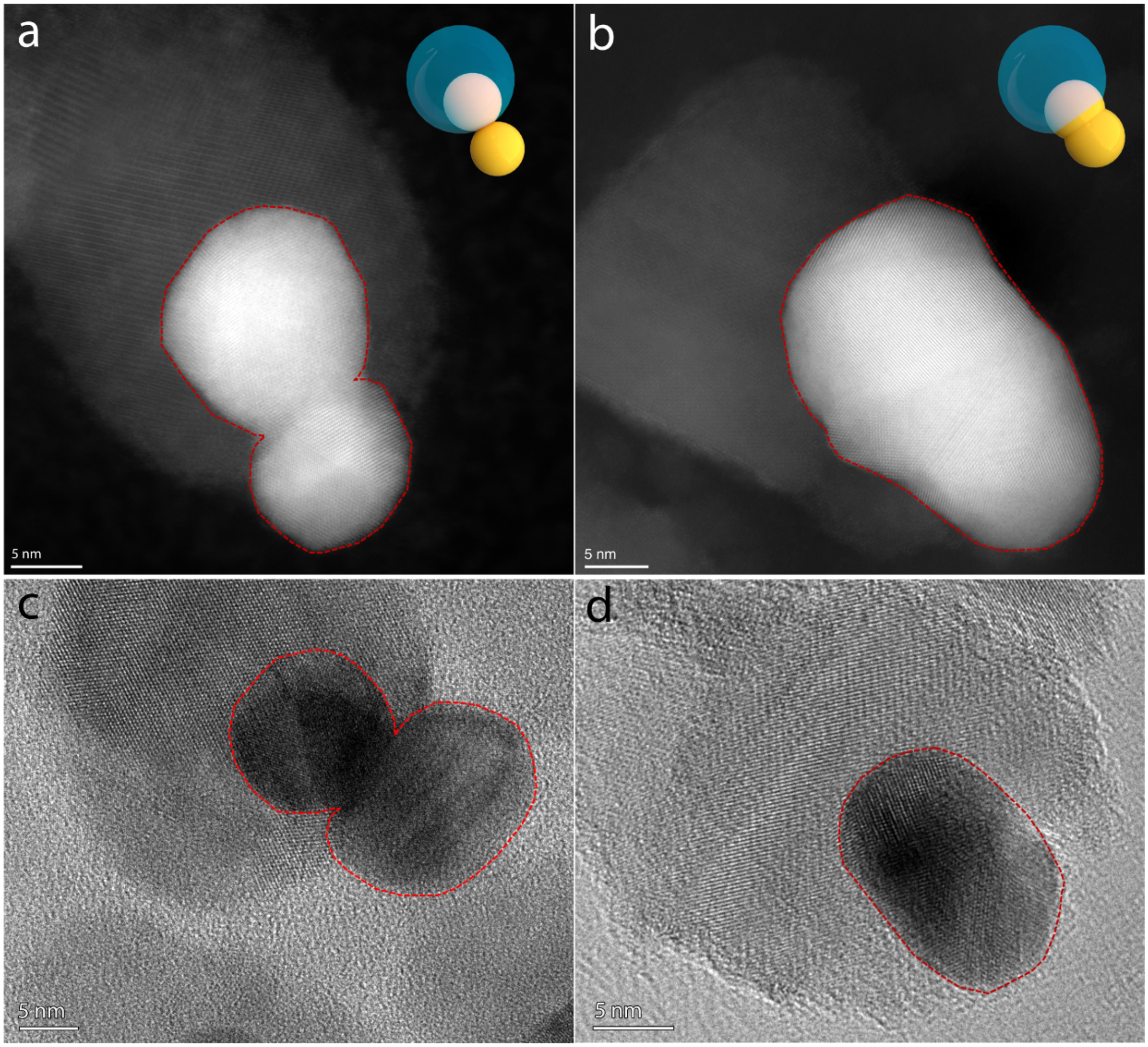

Figure S8. Comparison between HR-(S)TEM images of (a, c) t-CZTS@Au-Au and (b, d) p-CZTS@Au-Au, showing the distinct degrees of singularity in the two hybrid nanostructures. The red dot lines depict the contours of the Au entities, showing the *calabash-like* morphology and the *wax gourd-like* morphology for the Au entity in t-CZTS@Au-Au and p-CZTS@Au-Au, respectively.

Note: From the synthetic perspectives, t-CZTS@Au-Au and p-CZTS@Au-Au were prepared from t-CZTS@Au and p-CZTS@Au, respectively. As shown in **Figure 2b** & **2c** and **Figure S12** & **S13**, t-CZTS@Au has a small opening of Au NP without being covered by CZTS, while p-CZTS@Au has a much larger one. Under the constraint of CZTS, the second Au NP, overgrown from the small

opening, in t-CZTS@Au-Au has thus a small interfacial contact with the first Au NP. By contrast, the second Au NP in p-CZTS@Au-Au has a much larger interfacial contact with the first Au NP. As a result, t/p-CZTS@Au-Au have distinct differences, especially in the *curvature* of the surfaces near the interface between the two Au NPs (please see the schemes in **Figure S8**). Such differences are also reflected by the distinct morphologies of the Au entity in t/p-CZTS@Au-Au (t-CZTS@Au-Au: *calabash-like* morphology *vs*. p-CZTS@Au-Au: *wax gourd-like* morphology), as revealed by the HR-(S)TEM images in **Figure S8**.

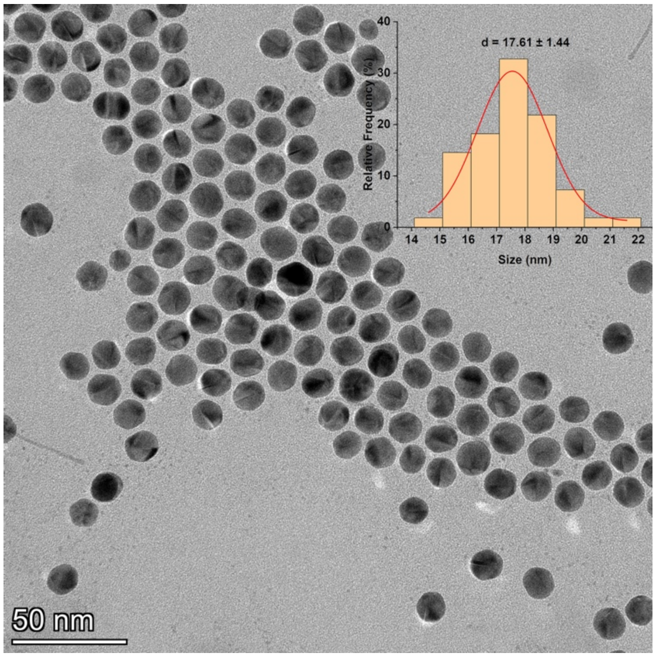

Figure S9. TEM image of the Au NPs studied in this work. The inset is the particle size distribution histogram showing an average size of ~18 nm.

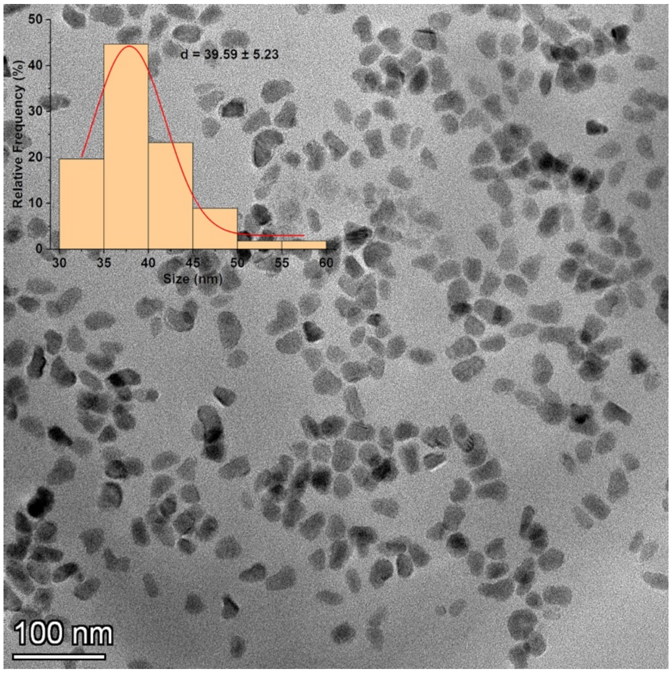

Figure S10. TEM image of the CZTS NPs studied in this work. The inset is the particle size distribution histogram showing an average size of ~40 nm.

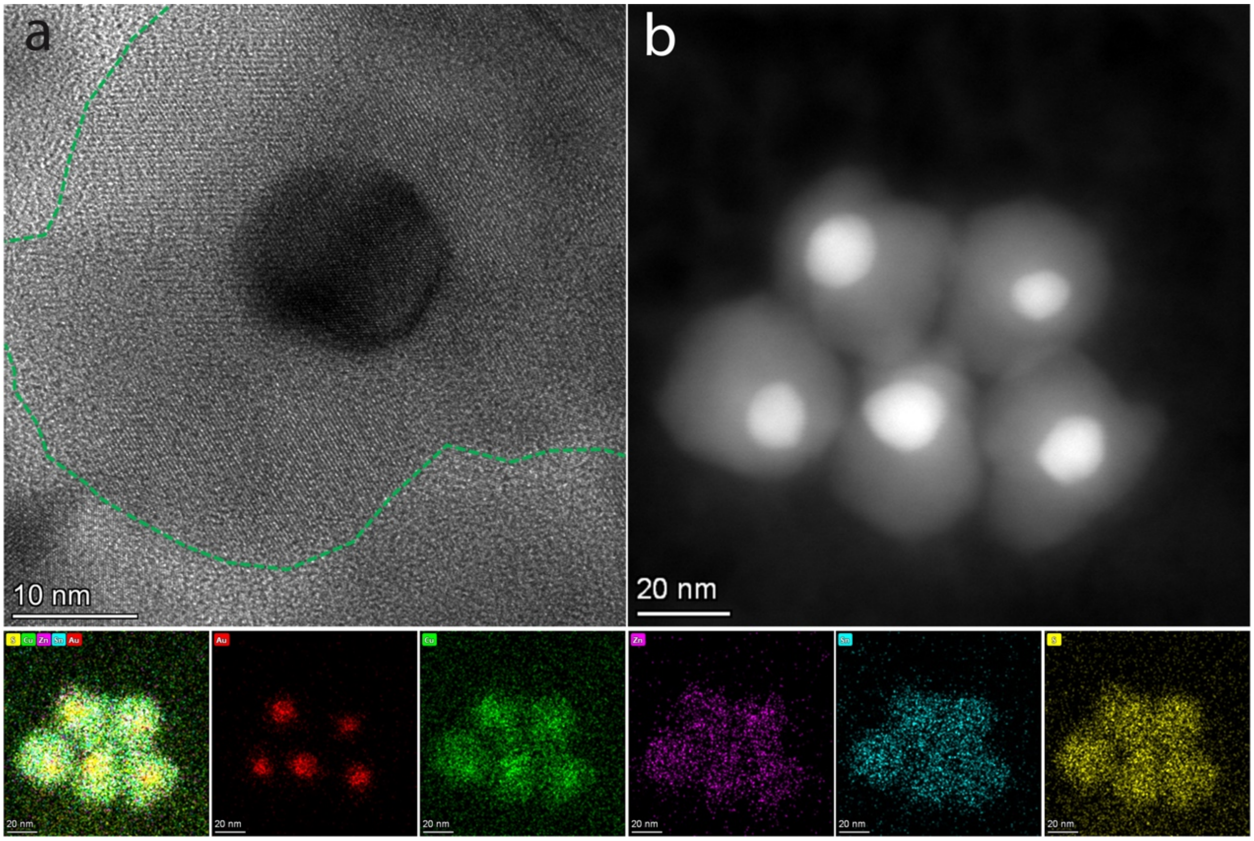

Figure S11. (a) HRTEM image and (b) HAADF-STEM image and the corresponding elemental mappings (bottom row) of f-CZTS@Au HNSs. The dot lines in (a) denote the perimeters of CZTS.

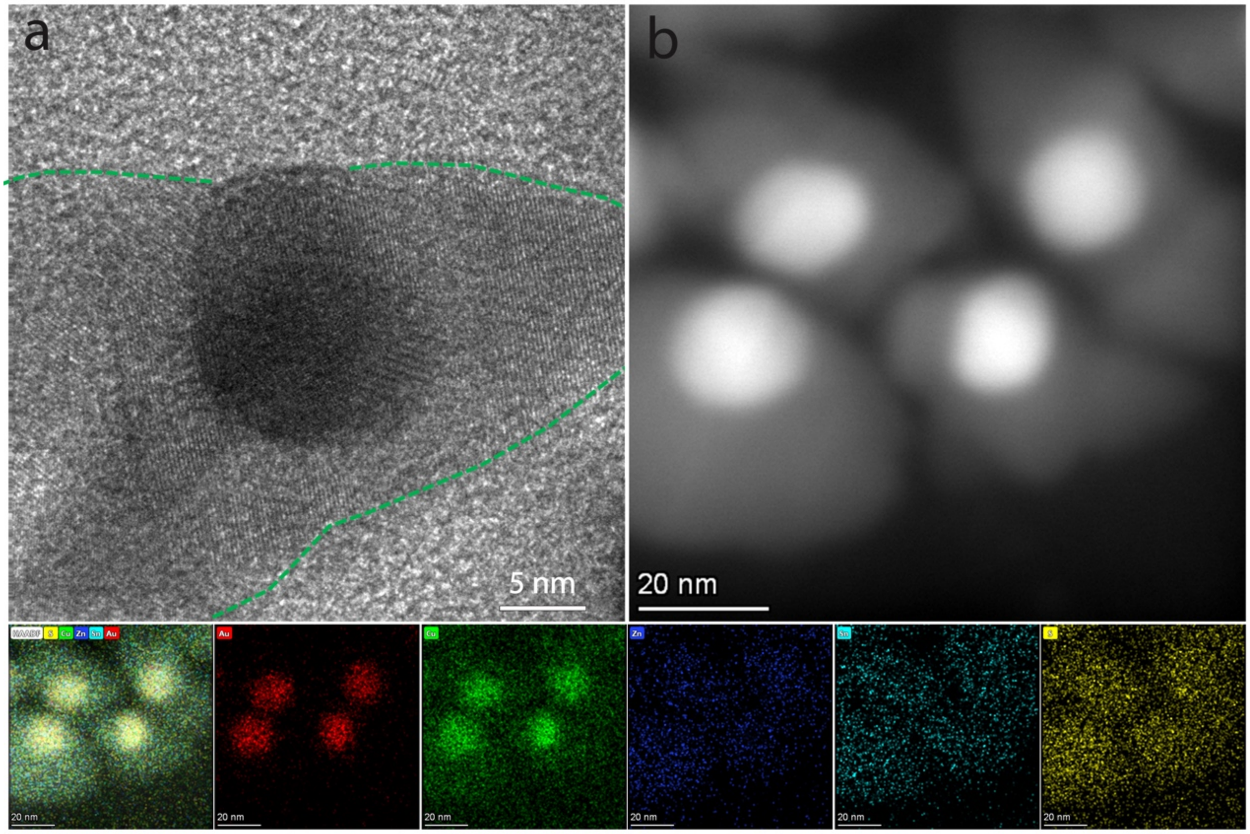

Figure S12. (a) HRTEM image and (b) HAADF-STEM image and the corresponding elemental mappings (bottom row) of t-CZTS@Au HNSs. The dot lines in (a) denote the perimeters of CZTS.

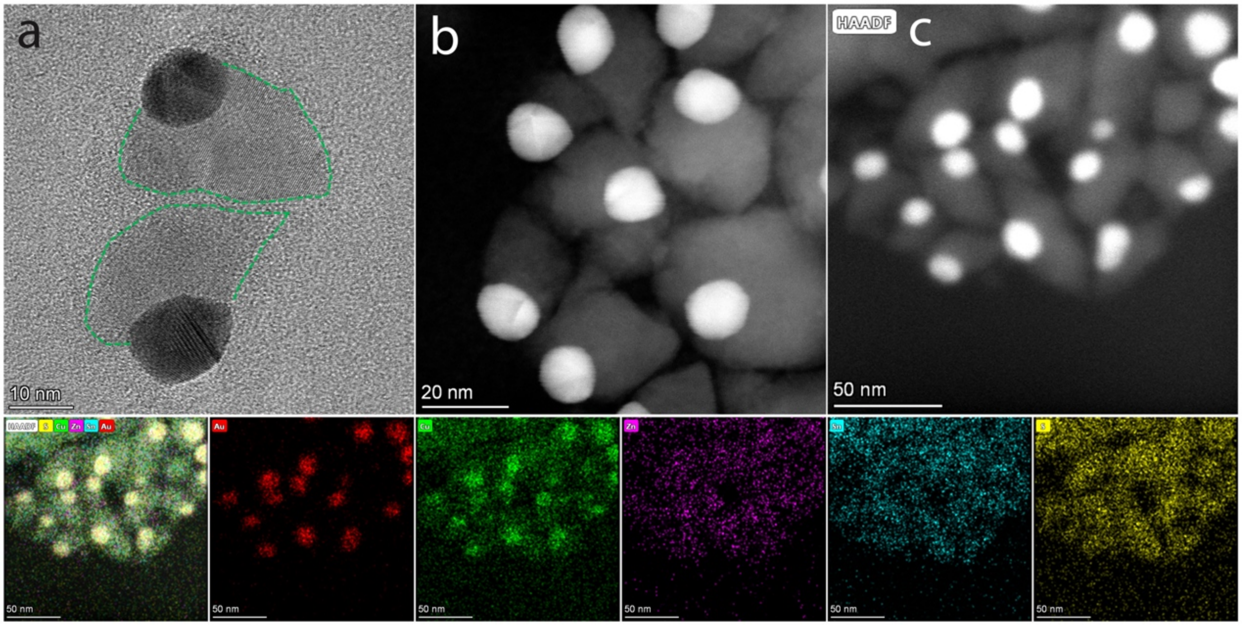

Figure S13. (a) HRTEM image, (b) HAADF-STEM image and (c) HAADF-STEM image and the corresponding elemental mappings (bottom row) of p-CZTS@Au HNSs. The dot lines in (a) denote the perimeters of CZTS.

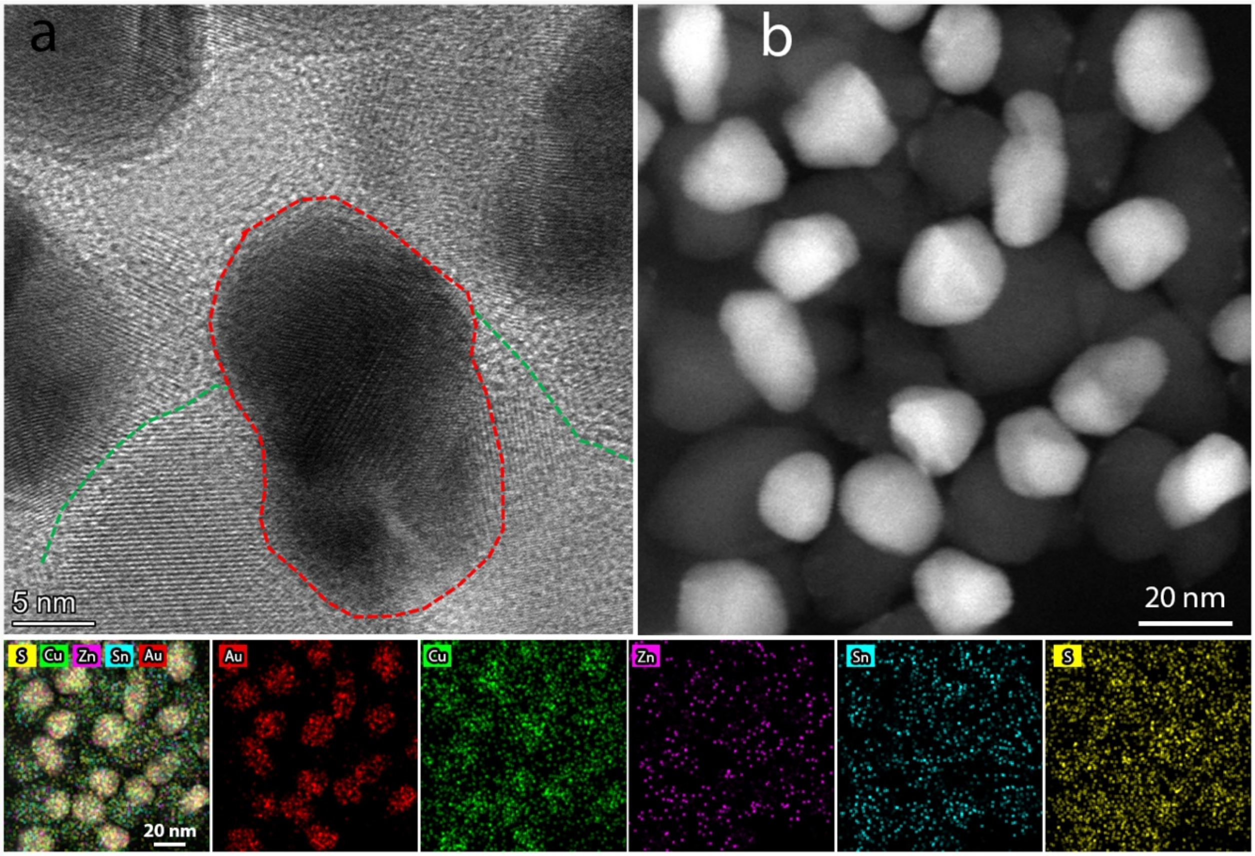

Figure S14. (a) HRTEM image and (b) HAADF-STEM image and the corresponding elemental mappings (bottom row) of p-CZTS@Au-Au HNSs. The red and green dot lines in (a) denote the perimeters of Au and CZTS component, respectively.

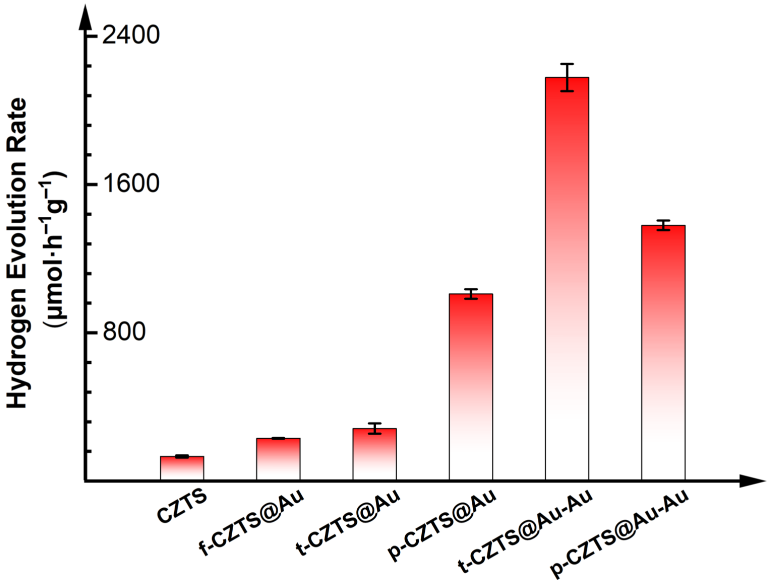

Figure S15. Photocatalytic hydrogen production rates normalized by the mass of only CZTS.

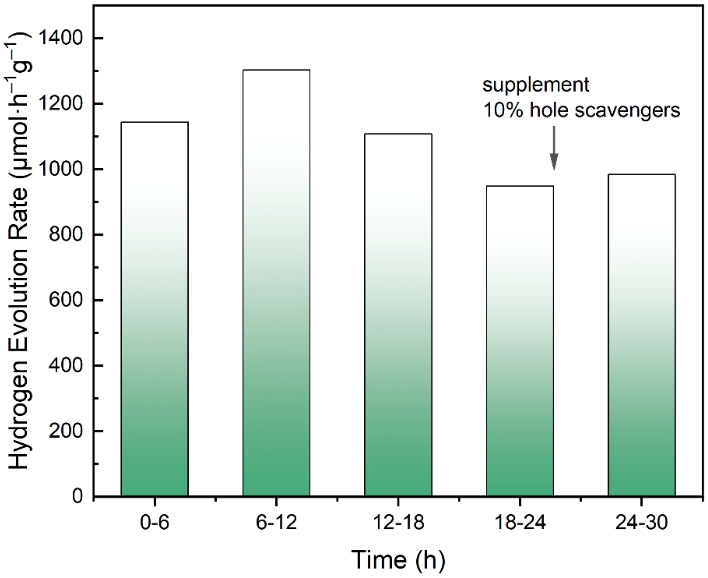

Figure S16. Recycle hydrogen generation property of t-CZTS@Au-Au. The slightly increased activity in the second cycle might be due to the decomposition of residual organic molecules at the nanoparticle surface that increased the catalyst active area. The slightly decreased activity with time in the third and fourth cycle was partly caused by the decreasing concentration of hole scavengers. When 10% of the hole scavengers was supplemented at the end of the fourth cycle, the activity partly recovered.

Photoelectrochemistry Study

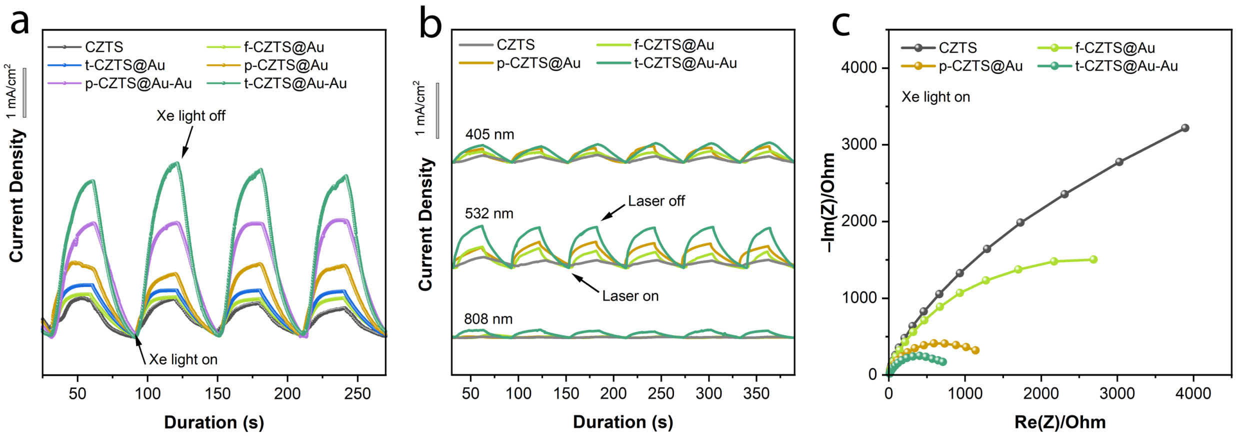

Figure S17. (a, b) photocurrent and (c) electrochemical impedance spectra of CZTS and Au/CZTS HNSs supported on a FTO-coated glass substrate under chopped Xe light (a), chopped laser (b) and continuous Xe light illumination (c), respectively.

Note: As suggested by earlier studies, the photoelectrons generated upon illumination participate in the water reduction over the active edge sites of the sulfide structure of CZTS, producing the hydrogen.[1] We employed photoelectrochemistry to study the carrier dynamics, as it is sensitive to probe the light response of the photocatalysts. First, the availability of photoelectrons, which is overall determined by the charge carrier photogeneration, separation and transport, is pivotal for efficient photocatalytic H_2 production.[19] We used the photocurrent produced from the photoelectrochemical reduction of ethyl viologen dipercholorate ($EV(ClO_4)_2$) as an indicator to evaluate the quantity of photogenerated excitons.[1] **Figure S17** reports the chronoamperometric responses of photocathodes made of the CZTS based photocatalysts under xenon light irradiation with periodic light chopping (see details in the experimental section). Again, all the Au/CZTS HNSs exhibit enhanced photocurrent density over CZTS and the enhancement follows the similar trend as observed in the photocatalytic hydrogen evolution rate, i.e., CZTS NPs < f-CZTS@Au < t-CZTS@Au < p-CZTS@Au < p-CZTS@Au-Au < t-CZTS@Au-Au. By conducting photocatalytic and photoelectrochemical tests with only visible light (> 400 nm), we demonstrate that both the UV light and the visible light contribute to the enhanced photocatalysis, despite that the former is more efficiently than the latter in driving the photocatalysis (**Figure S18**). We further recorded the current density of photocathodes made of CZTS NPs, f-CZTS@Au, p-CZTS@Au

and t-CZTS@Au-Au under the laser excitation of three monochromatic wavelengths (i.e., 405, 532 and 808 nm) (**Figure S17b**). Both the samples and excitation wavelengths were chosen based on their distinct and plasmonic characteristics as displayed in the extinction spectra (**Figure 3a**). While we note that the laser-induced thermal effect may play a non-negligible role in the photocurrents (as reflected by the slow current transient),[20] under all studied circumstances, t-CZTS@Au-Au shows the highest response to light excitation, agreeing well with its broadband and intense absorption. By contrast, f-CZTS@Au and p-CZTS@Au exhibit marked enhancement of current density over CZTS only at the wavelength of 405 and 532 nm. It is noteworthy that a noticeable light response is still observed for t-CZTS@Au-Au at 808 nm, though much weaker than at 405 and 532 nm. The weaker photocurrent density at 808 nm is partly due to the lower absorption of t-CZTS@Au-Au in the near-IR range that decreases the population of the plasmonic hot electrons. In addition, the intrinsic low kinetic energy of the plasmonic hot electrons due to the excitation by the long-wavelength (808 nm) and thus low-energy (1.53 eV) photons can be another key reason. Notwithstanding the weaker photocurrent, the t-CZTS@Au-Au sample shows an impressive enhancement over the pure CZTS sample that produces nearly no photocurrent. With regard to p-CZTS@Au, it presents a higher current density than f-CZTS@Au does at both 405 and 532 nm (**Figure S17b**), but lower at 808 nm (**Figure S19**), which is likely because the two wavelengths 405 and 532 nm are closer to the SPR band of the former while 808 nm to the latter. Finally, electrochemical impedance spectra (EIS), wherein the diameter of the semicircle in the Nyquist plot is positively correlated with the charge transfer resistance at the electrode-electrolyte interface, indicate that t-CZTS@Au-Au under illuminated conditions improved much more remarkably the charge transfer kinetics than p-CZTS@Au and f-CZTS@Au over the pure CZTS (**Figure S17c**), assumably because of the much shortened charge diffusion length and/or higher chemical potential of charges enabled by the unique singular structure. Overall, these results verify that LSPR could promote the photoelectrons for chemical reactions.

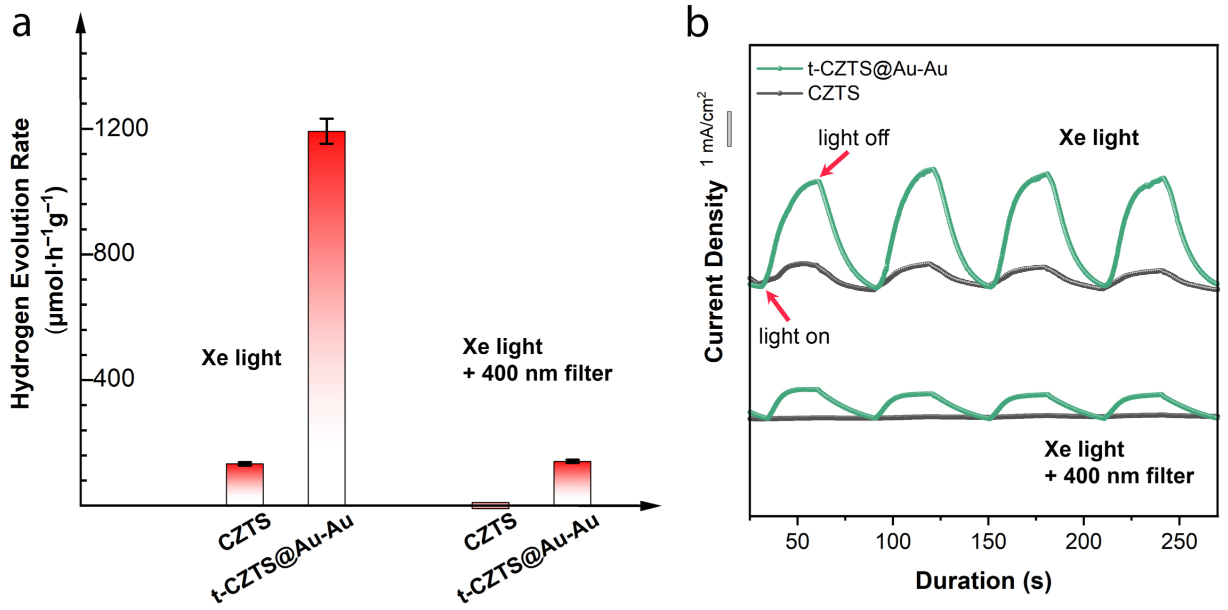

Figure S18. (a) Photocatalytic and (b) photoelectrochemical tests with Xe light and visible light which was obtained by adding a 400 nm-filter to the Xe light. In (a), CZTS under the excitation of visible light shows too trivial hydrogen evolution to be detected by a gas chromatography.

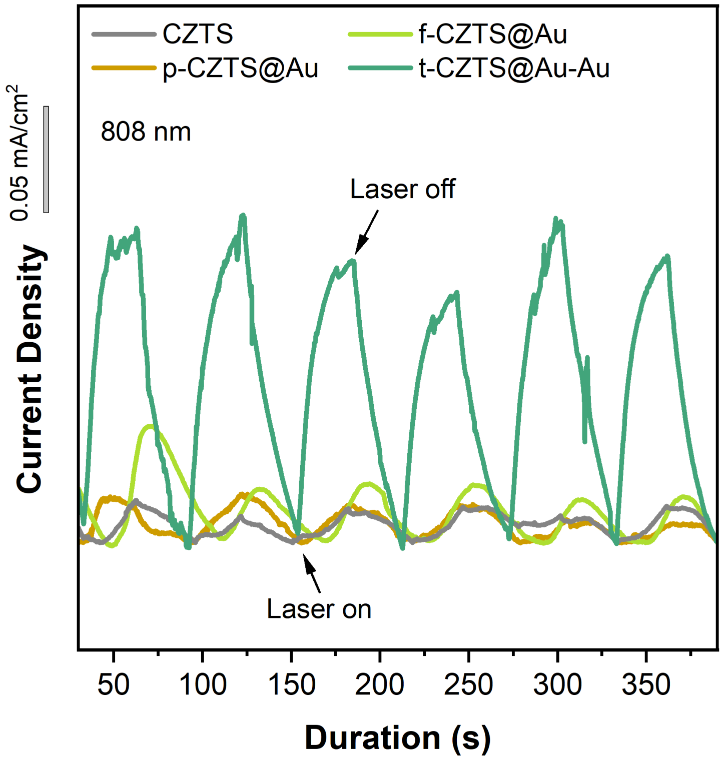

Figure S19. Photocurrent of CZTS and Au/CZTS HNSs supported on a FTO-coated glass substrate under chopped laser of 808 nm. Note that this figure shows an enlarged view of the light response under the excitation of 808 nm laser presented in Figure S17b.

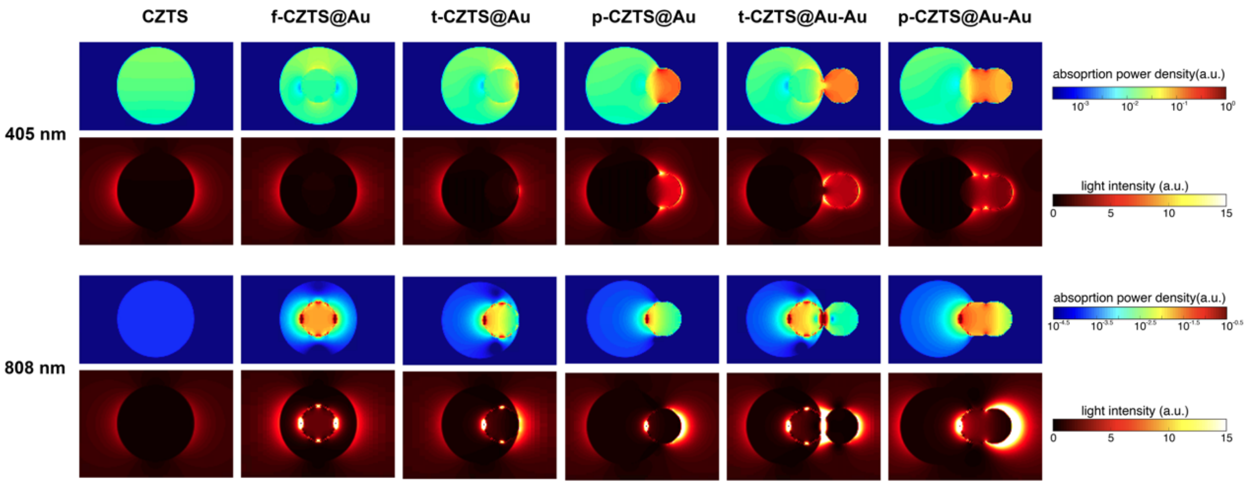

Figure S20. The FDTD simulated spatial distribution of absorption power density (Row 1 and 3, from top to bottom) and light intensity (Row 2 and 4, from top to bottom) at 405 nm (Row 1 and 2, from top to bottom) and 808 nm (Row 3 and 4, from top to bottom) of CZTS, f-CZTS@Au, t-CZTS@Au, p-CZTS@Au, t-CZTS@Au-Au and p-CZTS@Au-Au.

Transformation Optics for Realistic Configurations

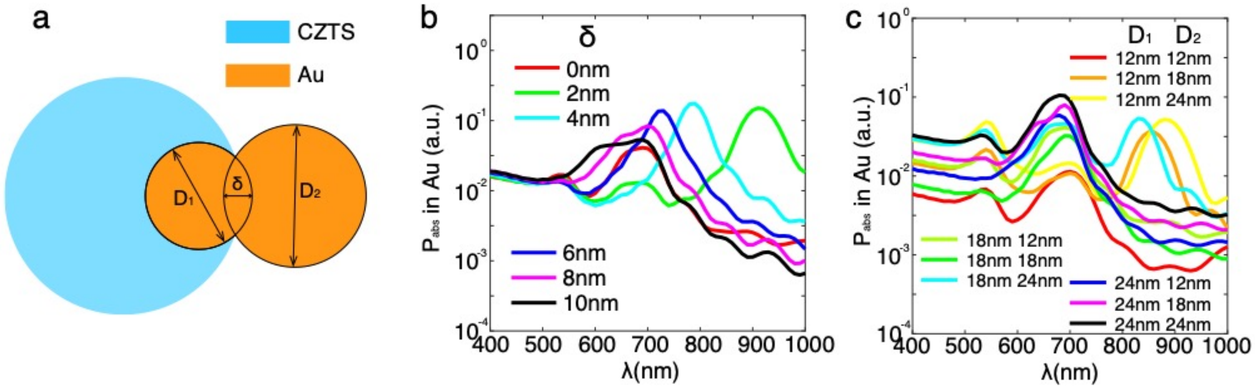

Figure S21. Absorption spectra for configurations with overlap and size fluctuations between the two nanoparticles. (a) A demonstration of the configuration, with $D_{1,2}$ being the diameters of the nanoparticles and δ being the overlap of the two nanoparticles. (b) The power consumption P_{abs} in Au for the configurations with different values of δ. (c) The power consumption P_{abs} in Au for the configurations with different values of D_1 and D_2.

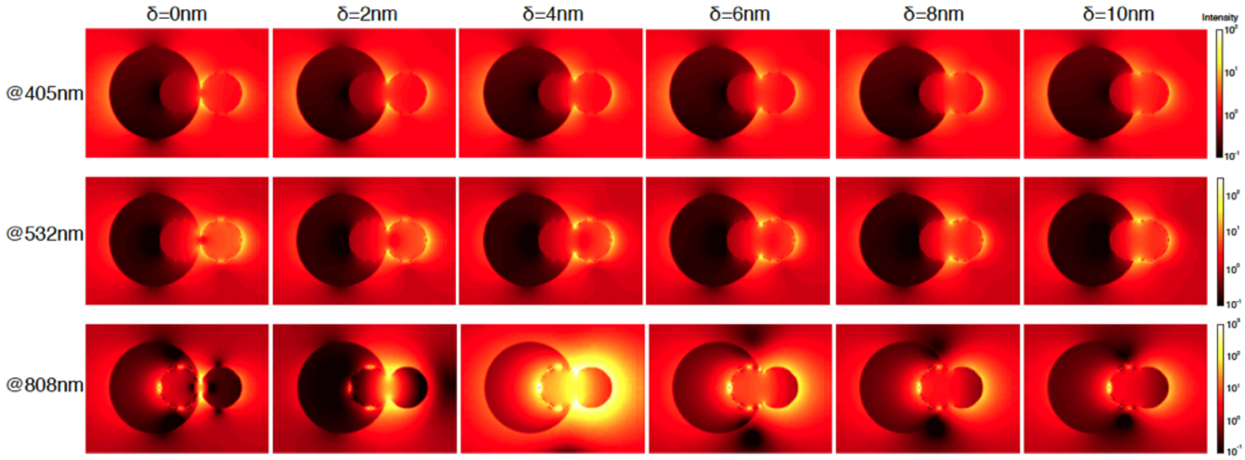

Figure S22. The adiabatic focusing for contacting nanospheres with different values of δ. The incident light wavelength varies from (a) 405 nm, (b) 532 nm to (c) 808 nm. Near-field enhancement at contact region is shown in cases with large overlap up to δ = 6-8 nm.

Note: In the manuscript, we model the contact between the Au nanoparticles as a point (singularity) and the size of the Au nanoparticles identical. In practical applications, point contacts (singularities) are unlikely to be realised due to limitations in synthesis and the surface tension of the metal. However, the bluntness of contacts can also be modelled by transformation optics,[21,22] demonstrating the absorption enhancement and adiabatic focusing at a broad spectral range. Meanwhile, the gigantic field enhancement effect on the kissing points always occurs with contacting spheres with different diameters.[23]

Besides the previous theoretical work, [21-23] here we numerically investigate the absorption spectra considering the parameters from practical geometries, as demonstrated in **Figure S21a**. We consider two factors; the overlap (δ) and the size difference (D_1, D_2) between the two contacting spheres. **Figure S21b** shows the absorption spectra in the two Au nanoparticles when the kissing point ($\delta = 0$) degrades to a contacting area ($\delta > 0$), while **Figure S21c** illustrates the cases of two Au nanoparticles with different sizes. A prominent absorption improve is realised compared with single Au nanoparticles.

Figure S21 also illustrates that size fluctuation may also play an important role in the broadband absorption achieved experimentally, together with the contacts predicted by transformation optics. However, the unique feature of adiabatic focusing of transformation optics guarantees the strong near-field enhancement around the contacting area at different wavelengths, in spite of the bluntness of the kissing point (cases with $\delta > 0$, shown in **Figure S22**). Photons at different wavelengths can be simultaneously concentrated at the same region, improving the generation rate of hot electrons. With our design, the area is also the tri-phase region where the chemical reaction takes place, maximising the effect of transformation optics for photocatalysis.

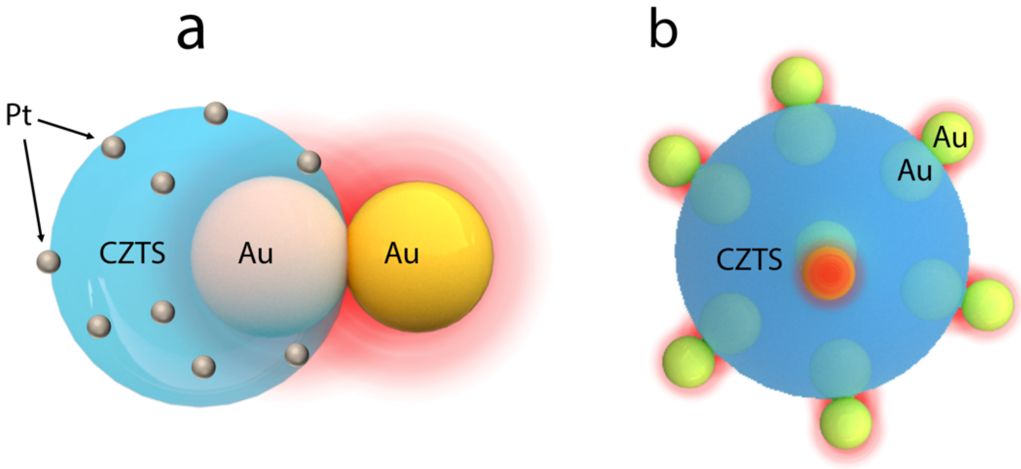

Figure S23. Conceptual strategies to further increase the performance of photocatalytic H_2 production of t-CZTS@Au-Au via (a) depositing Pt nanoparticles on the CZTS nanoparticle surface and (b) increasing the number density of Au-Au dimers at the CZTS nanoparticle.

Note: The apparent quantum efficiency (AQE) of t-CZTS@Au-Au under the present conditions is 0.044% (see the calculation in the experimental section), which does not show advantages with respect to nowadays the state-of-the-art photocatalytic systems.[24] Nevertheless, since this work focuses substantially on studying how the optical geometric structure fundamentally affects the photocatalytic activity, whereas the AQE can be easily raised by simply increasing the amount of the t-CZTS@Au-Au particles in the photocatalysis, the further enhancement of the photocatalytic performance from the perspectives of optimizing the catalyst design would be more intriguing. In this sense, by increasing the density of the plasmonic singularity through incorporating more Au-Au dimers at the same single CZTS NP or the sharpness of plasmonic singularity via more meticulous chemistry, a further enhancement in the photocatalytic performance can be expected. Moreover, considering Pt is a prominent H_2-evolution catalyst, deposition of Pt NPs onto the CZTS surface of the t-CZTS@Au-Au hybrid nanostructures also holds the potential to additionally elevate the hydrogen production rate (see **Figure S23** for conceptual schemes).

3. Supporting References

(1) Ha, E.; Lee, L. Y.; Wang, J.; Li, F.; Wong, K. Y.; Tsang, S. C. Significant enhancement in photocatalytic reduction of water to hydrogen by Au/Cu_2ZnSnS_4 nanostructure. *Adv. Mater.* **2014,** *26*, (21), 3496-500.

(2) Yu, X.; Shavel, A.; An, X.; Luo, Z.; Ibanez, M.; Cabot, A. Cu_2ZnSnS_4-Pt and Cu_2ZnSnS_4-Au Heterostructured Nanoparticles for Photocatalytic Water Splitting and Pollutant Degradation. *J. Am. Chem. Soc.* **2014,** *136*, (26), 9236-9239.

(3) Johnson, P. B.; Christy, R. W. Optical Constants of the Noble Metals. *Phys. Rev. B* **1972,** *6*, (12), 4370-4379.

(4) Kedenburg, S.; Vieweg, M.; Gissibl, T.; Giessen, H. Linear refractive index and absorption measurements of nonlinear optical liquids in the visible and near-infrared spectral region. *Opt. Mat. Express* **2012,** *2*, 1588-1611.

(5) Zhao, H.; Persson, C. Optical properties of Cu(In,Ga)Se_2 and $Cu_2ZnSn(S,Se)_4$. *Thin Solid Films* **2011,** *519*, (21), 7508-7512.

(6) Wang, X.; Ruan, Y.; Feng, S.; Chen, S.; Su, K. Ag Clusters Anchored Conducting Polyaniline As Highly Efficient Cocatalyst for Cu_2ZnSnS_4 Nanocrystals toward Enhanced Photocatalytic Hydrogen Generation. *ACS Sustain. Chem. Eenergy* **2018,** *6*, (9), 11424-11432.

(7) Yu, X.; An, X.; Genc, A.; Ibanez, M.; Arbiol, J.; Zhang, Y.; Cabot, A. Cu_2ZnSnS_4-PtM (M = Co, Ni) Nanoheterostructures for Photocatalytic Hydrogen Evolution. *J. Phys. Chem. C* **2015,** *119*, (38), 21882-21888.

(8) Yuan, M.; Wang, J.-L.; Zhou, W.-H.; Chang, Z.-X.; Kou, D.-X.; Zhou, Z.-J.; Tian, Q.-W.; Meng, Y.-N.; Zhou, Y.-M.; Wu, S.-X. Cu_2ZnSnS_4-CdS heterostructured nanocrystals for enhanced photocatalytic hydrogen production. *Catal. Sci. Technol.* **2017,** *7*, (18), 3980-3984.

(9) Sun, K.; Zhao, X.; Zhang, Y.; Wu, D.; Zhou, X.; Xie, F.; Tang, Z.; Wang, X. Enhanced photocarrier separation in novel Z-scheme Cu_2ZnSnS_4/Cu_2O heterojunction for excellent photocatalyst hydrogen generation. *Mater. Chem. Phys.* **2020,** *251* 123172.

(10) Jiang, F.; Pan, B.; You, D.; Zhou, Y.; Wang, X.; Su, W. Visible light photocatalytic H_2 production activity of epitaxial Cu_2ZnSnS_4/ZnS heterojunction. *Catal. Commun.* **2016,** *85*, 39-43.

(11) Gogoi, G.; Arora, S.; Vinothkumar, N.; De, M.; Qureshi, M. Quaternary semiconductor Cu_2ZnSnS_4 loaded with MoS_2 as a co-catalyst for enhanced photo-catalytic activity. *RSC Adv.* **2015,** *5*, (51), 40475-40483.

(12) Hu, Y.; Sun, Y. A Generic Approach for the Synthesis of Dimer Nanoclusters and Asymmetric Nanoassemblies. *J. Am. Chem. Soc.* **2013**, *135* (6), 2213-2221.

(13) Wang, Z.; He, B.; Xu, G.; Wang, G.; Wang, J.; Feng, Y.; Su, D.; Chen, B.; Li, H.; Wu, Z.; et al. Transformable masks for colloidal nanosynthesis. *Nat. Commun.* **2018**, *9* (1), 563.

(14) Gilroy, K. D.; Peng, H. C.; Yang, X.; Ruditskiy, A.; Xia, Y. Symmetry breaking during nanocrystal growth. *Chem. Commun.* **2017**, *53* (33), 4530-4541.

(15) Sun, Y. G. Interfaced heterogeneous nanodimers. *Natl. Sci. Rev.* **2015**, *2* (3), 329-348.

(16) Huang, J.; Mensi, M.; Oveisi, E.; Mantella, V.; Buonsanti, R. Structural Sensitivities in Bimetallic Catalysts for Electrochemical CO_2 Reduction Revealed by Ag-Cu Nanodimers. *J. Am. Chem. Soc.* **2019**, *141* (6), 2490-2499.

(17) Huang, J.; Zhu, Y.; Liu, C.; Shi, Z.; Fratalocchi, A.; Han, Y. Unravelling Thiol's Role in Directing Asymmetric Growth of Au Nanorod-Au Nanoparticle Dimers. *Nano Lett.* **2016**, *16* (1), 617-623.

(18) Feng, J.; Yang, F.; Wang, X.; Lyu, F.; Li, Z.; Yin, Y. Self-Aligned Anisotropic Plasmonic Nanostructures. *Adv. Mater.* **2019**, *31* (19), e1900789.

(19) Yu, X.; An, X.; Genç, A.; Ibáñez, M.; Arbiol, J.; Zhang, Y.; Cabot, A. Cu_2ZnSnS_4–PtM (M = Co, Ni) Nanoheterostructures for Photocatalytic Hydrogen Evolution. *The Journal of Physical Chemistry C* **2015**, *119* (38), 21882-21888.

(20) Zhan, C.; Liu, B. W.; Huang, Y. F.; Hu, S.; Ren, B.; Moskovits, M.; Tian, Z. Q. Disentangling charge carrier from photothermal effects in plasmonic metal nanostructures. *Nat. Commun.* **2019**, *10* (1), 2671.

(21) Luo, Y.; Lei, D. Y.; Maier, S. A. & Pendry, J. B. Broadband light harvesting nanostructures robust to edge bluntness. *Phys. Rev. Lett.*, **2012**, 108(2), 023901.

(22) Luo, Y.; Lei, D. Y.; Maier, S. A. & Pendry, J. B. Transformation-optics description of plasmonic nanostructures containing blunt edges/corners: from symmetric to asymmetric edge rounding. *ACS nano,* **2012**, 6(7), 6492-6506.

(23) Aubry, A.; Lei, D. Y.; Maier, S. A. & Pendry, J. B. Interaction between Plasmonic Nanoparticles Revisited with Transformation Optics. *Phys. Rev. Lett.* **2010**, 105 (23), 200901.

(24) Zhou, P.; Navid, I. A.; Ma, Y.; Xiao, Y.; Wang, P.; Ye, Z.; Zhou, B.; Sun, K.; Mi, Z. Solar-to-hydrogen efficiency of more than 9% in photocatalytic water splitting. *Nature* **2023**, *613* (7942), 66-70.
